# CKB inhibits epithelial-mesenchymal transition and prostate cancer progression by sequestering and inhibiting AKT activation

**DOI:** 10.1016/j.neo.2021.09.005

**Published:** 2021-10-24

**Authors:** Zheng Wang, Mohit Hulsurkar, Lijuan Zhuo, Jinbang Xu, Han Yang, Samira Naderinezhad, Lin Wang, Guoliang Zhang, Nanping Ai, Linna Li, Jeffrey T. Chang, Songlin Zhang, Ladan Fazli, Chad J. Creighton, Fang Bai, Michael M. Ittmann, Martin E. Gleave, Wenliang Li

**Affiliations:** a Texas Therapeutics Institute, Brown Foundation Institute of Molecular Medicine, University of Texas Health Science Center at Houston, Houston, TX, USA; b University of Texas MD Anderson Cancer Center UTHealth Graduate School of Biomedical Sciences, Houston, TX, USA; c Department of Pathology, School of Basic Medical Sciences of Fujian Medical University, Fuzhou, China; d Fujian Maternity and Child Health Hospital, Affiliated Hospital of Fujian Medical University, Fuzhou, China; e Shanghai Institute for Advanced Immunochemical Studies, School of Life Science and Technology, ShanghaiTech University, Shanghai, China; f Department of Clinical Laboratory, Shanghai Jiao Tong University Affiliated Sixth People's Hospital, Shanghai, China; g Department of Integrative Biology and Pharmacology, School of Medicine, University of Texas Health Science Center at Houston, Houston, TX, USA; h Department of Pathology and Laboratory Medicine, University of Texas Health Science Center at Houston, Houston, TX, USA; i Department of Urologic Sciences and Vancouver Prostate Centre; University of British Columbia, Vancouver, British Columbia, Canada; j Department of Medicine and Dan L. Duncan Comprehensive Cancer Center, Baylor College of Medicine, Houston, TX, USA; k Department of Pathology and Immunology, Baylor College of Medicine, and Michael E. DeBakey VAMC, Houston, TX, USA

**Keywords:** Creatine kinase B (CKB), AKT activation, Epithelial-mesenchymal transition (EMT), Prostate cancer progression and metastasis, Physical sequestration and inhibition of AKT activation

## Abstract

Epithelial-mesenchymal transition (EMT) contributes to tumor invasion, metastasis and drug resistance. AKT activation is key in a number of cellular processes. While many positive regulators for either EMT or AKT activation have been reported, few negative regulators are established. Through kinase cDNA screen, we identified brain-type creatine kinase (CKB or BCK) as a potent suppressor for both. As a ubiquitously expressed kinase in normal tissues, CKB is significantly downregulated in several solid cancer types. Lower CKB expression is significantly associated with worse prognosis. Phenotypically, CKB overexpression suppresses, while its silencing promotes, EMT and cell migration*,* xenograft tumor growth and metastasis of prostate cancer cells. AKT activation is one of the most prominent signaling events upon CKB silencing in prostate cancer cells, which is in line with prostate cancer TCGA data. EMT enhanced by CKB silencing is abolished by AKT inhibition. Mechanistically, CKB interacts with AKT and sequestrates it from activation by mTOR. We further elucidated that an 84aa fragment at C-terminus of CKB protein interacts with AKT's PH domain. Ectopic expression of the 84aa CKB fragment inhibits AKT activation, EMT and cell proliferation. Interestingly, molecular dynamics simulation on crystal structures of AKT and CKB independently demonstrates that AKT's PH domain and CKB's 84aa fragment establish their major interaction interface. In summary, we have discovered CKB as a negative regulator of EMT and AKT activation, revealing a new mode of their regulation . We have also demonstrated that CKB downregulation is a poor prognosticator, which is sufficient to promote prostate cancer progression.

## Highlights

• Through unbiased kinome cDNA screening, we have identified creatine kinase brain-type (CKB) as a critical negative regulator of epithelial-mesenchymal transition .
• CKB is expressed significantly lower in tumors than in normal tissues and its downregulation correlates with poor prognosis in several solid cancer types.
• Its ectopic expression blocks, while its silencing promotes, AKT activation and prostate cancer progression.
• CKB physically sequestrates AKT from interacting with mTORC2 complex.
• We have also mapped the CKB and AKT interaction domains and further pinpointed a small fragment of 84aa on C-terminus of CKB protein as the key element in blocking AKT activation and Epithelial-mesenchymal transition.

## Introduction

Metastasis accounts for >90% of cancer death [1−3]. Identifying new regulators for cancer metastasis is critical for developing new and effective therapeutics to treat cancer patients and extend their lives. In tumors of epithelial origin, aberrant induction of epithelial-mesenchymal transition (EMT) contributes to tumor invasion and metastasis [[4], [5], [6]]. Increasing evidence indicates EMT also enables therapeutic resistance and tumor recurrence [7,8]. Like many biological processes, EMT is controlled by both positive and negative regulators [9]. EMT transcription factors (EMT-TFs), such as Twist1, Twist2, Snail, Slug, Zeb1 and Zeb2, are the most well-established regulators of EMT. They coordinately and collaboratively promote EMT. Other positive EMT regulators include the PI3K/AKT pathways, TGF beta, the hypoxia/HIF pathway, and many others [[9], [10], [11], [12]]. On the other hand, only a few negative regulators of EMT have been reported, such as the miR-200 family, PTEN and several long noncoding RNAs [[12], [13], [14], [15]].

To identify novel regulators and potential drug targets for EMT, we carried out a human kinase cDNA screen, from which we validated and reported 2 new EMT promoting kinases, CDKL2 and ZAK [16,17]. Our screen also uncovered the opposing side of the yin-yang regulation of EMT. We identified several candidates as EMT negative regulators. Among them, we chose to focus on CKB (creatine kinase, brain-type), because its downregulation in multiple solid cancer types is strikingly consistent as found in multiple public cancer genomics and proteomics datasets.

CKB, a ubiquitously expressed kinase, is best known to reversibly catalyze phosphate transfer between ATP and creatine phosphate [18]. It plays a central role in energy supply in tissues with large demand [19]. In spite of many datasets showing CKB downregulation in different cancers, the role of CKB in cancer progression has not been extensively studied. CKB has been reported to contribute to colon cancer progression and liver metastasis, with discrepancies in different studies [[20], [21], [22]]. Mello et al reported that CKB is downregulated, and its promoter is methylated in gastric cancer [23]. However, CKB's role in other cancer types and its cancer-associated mechanisms, in general, are largely unknown.

AKT is a key regulator for EMT, cell survival, growth, migration, polarity, metabolism, muscle and cardiomyocyte contractility, angiogenesis, and self-renewal of stem cells [24,25]. Not surprisingly, AKT dysfunction has been implicated in diverse pathological conditions, including cancer, developmental syndromes, overgrowth, cardiovascular disease, insulin resistance and type 2 diabetes, inflammatory and autoimmune disorders, and neurological disorders [24]. AKT activation in cancers is driven mainly by loss of function of PTEN, PI3K activation mutations, or RTK signaling [24,26]. Besides PTEN, few other well-known negative regulators of AKT activation have been reported, such as phosphatases INPP4B [27], SHIP [28,29] and PIB5PA [30].

In our study, we set out to investigate the role of CKB in prostate cancer progression and its mechanism of action. We found that CKB downregulation promotes AKT activation, EMT and prostate cancer progression. Of note, CKB inhibits EMT and prostate cancer progression by sequestrating AKT's interaction with and activation by mTORC2 complex. We have also mapped their interacting domains and pinpointed a small CKB protein fragment as the key domain for its blocking of EMT and AKT activation.

## Materials and methods

### Cell culture

The PC3 cells and LNCaP-derived LN3 cells used in this study were kindly provided by Dr. Isaiah Fidler as previously described [31,32], and were confirmed to match to PC3 and LNCaP cells from ATCC, respectively, by DNA STR fingerprinting (Biosynthesis Inc). 293T, LNCaP, VCaP, and DU145 cells were purchased from ATCC. PC3, LNCaP, VCaP, LN3 and DU145 cells were cultured in RPMI 1640 media, supplemented with 10% FBS and 1% penicillin-streptomycin. The 293T cells were cultured in DMEM with 10% FBS. All cell lines were routinely confirmed to be mycoplasma-free using MycoAlert Detection kit (#LT07-218, Lonza).

### Vector constructs and virus preparation

To generate all expression plasmids, PCR was performed following the manufacturer's instruction of Q5 High-Fidelity PCR Kit (#E0555S, New England Biolabs) by mixing templates and primers listed below. PCR products were purified by gel extraction kit (#L00418, Genescript) and digested with restriction enzymes (New England Biolabs). Ligation was performed using T4 DNA ligase (#M0202, New England Biolabs) and products were transformed in NEB Stable competent cells (#C3040, New England Biolabs, for viral vectors) and NEB 5-alpha competent cells ((#C2987, New England Biolabs, for nonviral vectors). Plasmids were extracted using GeneJET plasmid miniprep (#K0503, Thermo Scientific) and midiprep kit (#FERK0481, Thermo Scientific). All plasmids were subjected for Sanger sequencing to validate sequences. To clone human CKB cDNA into 3xflag-expression plasmid (pcDNA3.1/hygro-flag vectors [33]) primers and their annealing temperature (Tm) are Fw: CGCGGATCCATGCCCTTCTCCAACAGCCACAACGCACTGAA, Rev: CCGCTCGAGTCATTTCTGGGCAGGCATGAGGTCGTCGATGG. Tm = 72. To generate truncated CKB plasmids, human 3xflag-CKB plasmid was used as template. The primers for CKB truncation cloning and their Tm values are listed in Supplementary Table 1. For GFP-AKT-PH cloning, GST-AKT was used as template and primers are Fw: GTTCTCGAGCTGTGGCTATTGTGAAGGAGGG, Rev: TTAGGATCCCTTGAGGCCGTCAGCCACA; Tm = 72. GST-AKT full length and truncations are gifted by Dr. Keqiang Ye [34].

### Preparation of retroviruses for cDNA overexpression and lentiviruses for shRNA knockdown

Procedures for generating retroviruses for GFP or CKB cDNA in pJP1563-blasticidin vector, and lentiviruses for Scramble control shRNA and CKB shRNAs in pLKO.1-puro vector were similar to those in our previous studies [31,[35], [36], [37]]. TRC clone IDs for shCKB-1 and shCKB-2 are TRCN0000006053 (later renamed as TRCN0000220652) and TRCN0000010991, respectively.

### Animal experiments

All animal studies followed protocols approved by the Animal Welfare Committee at University of Texas Health Science Center at Houston. We have complied with all relevant ethical regulations. PC3 and DU145 cells expressing firefly luciferase were infected with retroviruses carrying either empty vector or CKB cDNA, or infected with lentiviruses carrying either control shRNA or shCKB. Stable CKB overexpression or knockdown cells were establish after antibiotics selection. For xenograft experiment, male 6 to 7 wk old NOD/SCID mice (Charles River Laboratories) were implanted with 1 to 2 million of cells per injection in 100 µl 1:1 of PBS and matrigel (subcutaneous injection), or in 50ul 1:1 of PBS and matrigel (prostate injection). Tumor growth were monitored by bioluminescence imaging at time points indicated in the figures. Mice were anaesthetized using isoflurane and injected intraperitoneally with 150 mg/kg luciferin in 100 µl (#119222, Caliper Life Sciences), and imaged using an IVIS Lumina II platform (Caliper Life Sciences) with Live Image software (Caliper Life Sciences). In each experiment, when a mouse with the biggest tumor reached the approved humane end point, all mice were sacrificed, the tumors were extracted and processed. Their lungs were also extracted and imaged ex vivo for metastasis by soaking in a culture plate with 300 µg/ml D-luciferin in PBS for 5 min.

### Generation of CRISPR/Cas9 CKB knockout constructs and KO cells

Target sequences for human CKB gene (GenBank accession: XM_017020952) were screened and selected using tools on crispr.mit.edu. Four target sequences and their guide RNAs were listed as follows. #1 target sequence on exon 2: GAAGCCGCTCGGCGTGCTCT (oligo1: CACCGGAAGCCGCTCGGCGTGCTCT; oligo2: AAACAGAGCACGCCGAGCGGCTTCC); #2 target sequence on exon 3: ATCATCGAGGACCGGCACGG (oligo1: CACCGATCATCGAGGACCGGCACGG; oligo2: AAACCCGTGCCGGTCCTCGATGATC); #3 target sequence on exon 3: CACCCGTACATCATGACCGT (oligo1: CACCGCACCCGTACATCATGACCGT; oligo2: AAACACGGTCATGATGTACGGGTGC); #4 target sequence on exon 1: CCCGCCCCCGCCCGGCCGCC (Oligo1: CACCGCCCGCCCCCGCCCGGCCGCC; oligo2: AAACGGCGGCCGGGCGGGGGCGGGC). gRNAs were cloned into PX458 construct following Feng Zhang lab's protocol (https://media.addgene.org/data/plasmids/52/52961/52961-attachment_B3xTwla0bkYD.pdf). Sequence of the insert in PX458 was verified by Sanger sequencing. PC3 cells were transfected with the plasmid DNA using TransIT-LT1 Transfection Reagent (#Mir2300, Mirus Bio). 48 h post transfection, cells were lysed and subjected to western blotting. For isolating single cell clones, PC3 cells were transfected with PX458 carrying #2 gRNA. 48 h later, cells were trypsinized and flow-sorted for GFP positive cells into 96-wells plate. Two weeks after single cells sorting, genomic DNAs at the target region were amplified by PCR (Fw: TCATCCAGACAGGCGTGGAC; Rev: GCTCATCGCTGGGCTTGTAG. Tm = 70) and the sequences of PCR products were determined by Sanger sequencing. Clone 7-2 with a 13bp deletion was selected for further study.

### Western blotting analysis

Cells were washed with ice-cold PBS supplemented with 10mM NaF and 2mM NaVO3 before adding lysis buffer (30mM Tris pH8.0, 20% glycerol, 200mM NaCl, 1.5mM MgCl2, 0.4mM EDTA, 1% NP-40, 1mM DTT, protease and phosphatase inhibitor, 0.5 mM PMSF) on ice for 30 min. Lysate were then sonicated by Branson Sonifier at 8 Watt for 10 s twice. After sonication, lysate was subjected to centrifuge at 4°C, 13,500 rpm for 30 min. Supernatant were transferred to a new tube and protein concentration were measured using BCA protein assay kit (#23225, ThermoFisher Scientific). Protein samples were then separated by SDS-PAGE and transferred to PVDF membrane. The membrane was blocked with 5% nonfat milk in TBST for 1 h at room temperature, followed by incubation of a primary antibody overnight at 4°C. Primary antibodies used were as follows: anti-Vimentin V9 (#ab8069, Abcam), anti-E-cadherin (#610181, BD Bioscience), anti-N-cadherin (#610920, BD Bioscience), anti-ZEB1 (#SC-25388, Santa Cruz Biotechnology), anti-ESRP1/2 (#210301C31, Rockland Immunochemicals), anti-Occludin (#SC-81812, Santa Cruz Biotechnology), anti-β-actin (#SC-68979, Santa Cruz Biotechnology), anti-CKB antibody (#ab125114; Abcam), anti-GAPDH antibody (#2118, Cell Signaling Technology), anti-p-AKT antibody (#4060, Cell Signaling Technology), anti-AKT antibody (#2920, Cell Signaling Technology), anti-SLUG antibody (#SC-166476, Santa Cruz Biotechnology), anti-TWIST antibody (#SC-81417 Santa Cruz Biotechnology), anti-Rictor antibody (#051471, Millipore sigma), anti-MTOR antibody (#2972, Cell Signaling Technology), anti-FLAG antibody (#8146, Cell Signaling Technology), anti-GST antibody (#SC-138, Santa Cruz Biotechnology). After washes, the membrane was incubated with HRP-linked anti-mouse IgG antibody (#7076, Cell Signaling Technology) or HRP-linked anti-rabbit IgG antibody (#7074, Cell Signaling Technology) for 1 h at room temperature. Membrane were then detected by Pierce ECL Western Blotting Substrate (#32106, Thermo Scientific) on autoradiography film.

### Immunohistochemistry (IHC) staining

Prostate cancer tissue microarray that contains 141 prostate tumors and 115 normal prostate tissues were obtained from Baylor College of Medicine and Biomax (#PR806, US Biomax). Tissue samples were obtained from the Human Tissue Acquisition and Pathology Core of the Dan L. Duncan Cancer Center at Baylor College of Medicine and were collected from fresh radical prostatectomy specimens after obtaining informed consent under an Institutional Review Board approved protocol. IHC staining procedure was described previously [16]. Briefly, slides with 5-μm sections of formalin-fixed, paraffin-embedded xenograft tumors were incubated with p-AKT primary antibody overnight at 4°C and HRP conjugated secondary antibody 1 h at room temperature. The immunohistochemistry reaction was developed with a DAB substrate Kit (#PK6104, Vector laboratories and #K8000, DAKO), slides were counterstained with hematoxylin and mounted in VectaMount permanent mounting medium (#H5000, Vector laboratories). For immunostaining, deparaffinized and dehydrated sections were washed for 5 min in PBS and treated with 3% hydrogenperoxide. Microwave antigen retrieval was done by placing the slide in 10 mm citrate buffer (pH 6.0) and microwaving on high power for 10 min. Slide was incubated in citrate buffer for a further 120 min at room temperature (RT), after which it was removed and rinsed by PBS. Vectastain elite ABC HRP kit (#PK7200, Vector Labs) was used for immunochemistry staining according to the manufacture's instruction. Anti-CKB antibody (#ab108388, Abcam) was used as first antibody at a dilution of 1:2000 at 4°C overnight. DAB reaction was terminated by soaking slide into PBS and mounting with VectaMount AQ Aqueous Mounting Medium (#H5501, Vector Labs). Images were acquired using ZEISS microscope and analyzed by Zen software at same parameters. Staining was scored using a multiplicative staining index of intensity (0–3) and extent (0–3) of staining yielding a 10-point staining index (0–9) as described previously [38]. Scores ranges from 0 to 3, 4 to 6 and 7 to 9 were described as weak, moderate and intense CKB expression, respectively. The significance of differences in CKB levels in normal vs tumor samples was assessed using Chi Square Test.

### Immunofluorescence assay

PC3 cells were infected with GFP-AKT-PH lentivirus to express AKT PH domain fusing with GFP and sorted by flow cytometry to select the GFP positive PC3-GFP-AKT-PH cells. Coverslips were placed in wells of a 12-well plate before seeding PC3-GFP-AKT-PH cells. 24 h later, 0.3ug of 3xflag tagged CKB full length or truncation plasmids were transfected into cells using TransIT-LT1 Transfection Reagent (#Mir2300, Mirus Bio). Twenty-four hours post transfection, cells were changed to no serum medium for 16 h before adding 100ng/ml EGF treatment for 5 min. For immunofluorescence assay, cells were washed once with ice-cold PBS and fixed immediately with 4% Paraformaldehyde (#J19943K2, ThermoFisher Scientific) for 15 min at room temperature (RT). Cells were permeabilized by adding 0.1% triton X-100 (Sigma). Nonspecific binding sites were blocked by 3% BSA (#J10867, ThermoFisher Scientific) for 1 h at RT. Mouse Anti-flag antibody(#8146, Cell Signaling Technology) was used at a dilution of 1:300 in blocking buffer at 4 °C overnight followed by incubation with Cy3-Goat anti-Mouse IgG Secondary Antibody (#NB7607, Novus Biologicals) for 2 h at RT. Nuclear were stained with Draq5 at a dilution of 1:1000 in dark chamber for 10 min. Cells were washed extensively in PBS between each incubation and mounted with ProLong Diamond Antifade Mountant solution. Images were pictured by Nikon eclipse TE2000-E microscope and analyzed by LAS AF lite software at same parameters. Image analysis and quantifications were performed using Image J software.

### Reverse transcription and quantitative PCR analysis

TRIzol (Invitrogen) was used to isolate RNA from cells. cDNA was generated by reverse transcription with iScript cDNA Synthesis Kit (Bio-Rad). Real time PCR was performed using SsoFast EvaGreen Supermix (Bio-Rad) in Bio-Rad CFX96 Real-Time PCR Detection System. The RT-qPCR primers are listed in Supplemental Table 1.

### Co-immunoprecipitation

For LNCaP, cells were washed twice with ice-cold PBS on ice before collecting and then lysed with lysis buffer (30mM Tris pH8.0, 20% glycerol, 200mM NaCl, 1.5mM MgCl2, 0.4mM EDTA, 1% NP-40, 1mM DTT) supplied with protease and phosphatase inhibitor (#88669, ThermoFisher Scientific) and 0.5mM PMSF on ice for 30 min. After that, lysate was centrifuge at 4°C, 13500rpm for 30 min. Supernatant were transferred to a new tube and protein concentration were measured using BCA protein assay kit (#23225, ThermoFisher Scientific). For PC3-1563-empty vector or PC3-CKB, cells were starved of serum for 16 h before adding 100ng/ml EGF (#CB40052, Corning) for 5 min. Cell lysate were collected as described above. Protein samples together with anti-AKT antibody (#2920, Cell Signaling Technology), anti-Rictor antibody (#051471, Millipore sigma) or mouse IgG isotype control antibody (#5415, Cell Signaling Technology) were incubated at 4°C overnight. To detect direct interaction of AKT and CKB in vitro, 2ug purified his-AKT1 (#PR3878D, Invitrogen) and his-CKB (#495H, Creative BioMart) were incubated at 4°C for 2h. Equal amount of anti-CKB antibody (#ab108388, Abcam) and rabbit IgG isotype control antibody (#3900, Cell Signaling Technology) were added for binding at 4°C overnight. A total of 80ul Protein A/G PLUS-Agarose slurry (#SC-2003, Santa Cruz Biotechnology) were washed with PBS for 3 times and added to each sample for binding at 4°C for 2h. Agarose beads were then washed with lysis buffer for 3 times followed by ice-cold PBS 3 times, and then mixed with 6x Laemmli sample buffer for 10 min at 95°C.

### GST pulldown assay

The 293T cells were transfected with GST-AKT full length or truncation plasmids together with 3xflag CKB full length or truncation plasmids. 36 h after transfection, cells were washed with ice-cold PBS and collected with lysis buffer supplied with protease and phosphatase inhibitor (#88669, ThermoFisher Scientific) and 0.5mM PMSF. 80ul glutathione sepharose 4B slurry (#17-0756-01, Millipore Sigma) were washed with PBS and added to each sample at 4°C on a rotating wheel for 3h. The beads were then washed with lysis buffer for 3 times and mixed with 6x Laemmli sample buffer for 10 min at 95°C.

### PIP3 pull down

His-tagged recombinant human AKT1 protein (#PR3878D, Invitrogen) were incubated with 0, 1 or 2ug his-tagged recombinant human CKB protein (#495H, Creative BioMart) at 4°C for 2 h on a rotating mixer. 40μL of PIP3 or control resin beads slurry (#PB345a, Echelon) were washed with PBS and added to each tube. Pull down reactions were performed at 4°C for 2 h on a rotating mixer. Beads were washed for 5 times with reaction buffer according to the manufacturer's instruction. Proteins were eluted by adding 2x Laemmli sample buffer for 10 min at 95°C, and then subjected to western blotting analysis.

### Dual-luciferase assay

The 1 × 10^4^ 293T cells were seeded in 96-well plate 24 h before transfection. 200ng 3xflag CKB full length or truncation plasmids together with 8ng Vimentin promoter luciferase reporter plasmid and 4ng pGL4.74 plasmids were transfected into cells using TransIT-LT1 Transfection Reagent (#Mir2300, Mirus Bio). Twenty-four to 36 h later, cells were lysed for luciferase detection using Pierce™ Renilla-Firefly Luciferase Dual Assay Kit (#16186, ThermoFisher Scientific) according to the manufacturer's instructions.

### Migration

Cells were cultured in medium without serum for 16 h before seeding into a 24-well transwell plate containing 8μm pore-size polycarbonate membrane inserts (#CLS3422, Corning Costar). 4 × 10^4^ cells were seeded and cultured in upper inserts in 150ul medium without serum, with 600ul full medium in lower chamber. Twenty-four hours later, nonmigrating cells were wiped from the upper side of the membrane. Then the membranes were fixed and stained with 0.5% crystal violet solution for further analysis. Image analysis and quantifications were performed using Image J software.

### Anchorage independent growth

PC3 cells carrying empty vector (Ctrl) or expressing CKB cDNA were seeded at 5 × 10^3^ cells per well of a 24-well Ultra Low Plate (Corning) and grown for 14 d in RPMI 1640 medium. Wells were fed every 3 d with 0.5 ml media. The spheres were imaged at day 14.

### Focus (colony) formation

Prostate cancer cells were seeded in 6-well plates at 500 or 1500 cells/well in duplicates or triplicates. Fresh RPMI media with 10% FBS were replenished every 5 to 6 d. After 10 or 14 d, the colonies were visualized and imaged by crystal violet staining. Image analysis and quantifications were performed using Image J software.

### Drug treatments and cell proliferation assay

PC3 parental, CKB shRNA or knockout cells were treated with DMSO (#276855, Sigma) or 25uM MK-2206 (#11593, Cayman Chemical) for 48h before collecting cell lysate. For cell proliferation assay, 600 cells were seeded into 96-well plate in RPMI 1640 culture medium containing 10% FBS. In the next day, medium was changed to RPMI 1640 supplemented with 1.25% FBS. Fluorescence was detected every 24 h using Alamar Blue reagent, incubating at 37°C for 4 h, then reading on a fluorescence plate reader (#Infinite M1000, Tecan) with excitation at 530nm and emission at 595nm.

### Genomics and proteomics data collections and analyses

As indicated, all datasets used in this study were downloaded from cBioPortal [39], Oncomine (http://www.oncomine.org), KM plotter (http://kmplot.com/analysis) or GEO database (http://www.ncbi.nlm.nih.gov/gds). The CPTAC data are from Chad Creighton and UALCAN (http://ualcan.path.uab.edu/analysis-prot.html). The transformed and normalized gene expression values from these sources were used in our analyses and statistical calculation.

### Statistical analyses

All data were plotted as Means  ±  SD. *P* values were obtained through 2-sided *t* test using GraphPad Prism or Microsoft Excel, unless otherwise indicated (either from public databases or using other statistical tests as specified). *P* < 0.05 was considered statistically significant.

### Predicting the candidate binding modes for CKB-AKT

Firstly, co-evolutionary analysis methods, Direct Coupling Analysis (DCA) [1,2] and RaptorX [3] were used to predict the possible interacting residue pairs between CKB and AKT. Top 20 pairs of co-varied residues were kept for each method. Then we randomly selected 2 pairs of residues, i.e., I103 and V106 for AKT, V350 and V354 for CKB, as observed in the 2 different co-evolutionary analyses, as the input of active site information for protein-protein docking. Two popular protein-protein docking methods, ZDOCK [40] and HADDOCK [41] were employed to predict the potential binding conformations for CKB and AKT, using the default parameters. Meanwhile, parallel protein-protein docking simulations without binding constraints, i.e., active site information, were also performed. In the docking process, the protein structure of CKB (PDB code: 3B6R) and AKT (PDB code: 6HHJ) were used as the starting structures. After collecting and clustering candidate binding poses, 3 major conformations were selected and used for further analysis.

### Determining the binding conformation for CKB-AKT

The full-length experimental structures of AKT was still not available. Structure of 6HHJ lacks the AKT C-terminal region of aa446-480. To make the prediction more reliable, protein structure modeling method I-TASSER [42] was used to construct the complete structure of AKT. Then, the binding complex of these 2 proteins was generated by superposing these 2 proteins to the complexes obtained from protein-protein docking simulations. The full-length binding complex were taken as the starting structure of molecular dynamics simulations by Desmond [43] with OPLS_2005 force filed [44]. Molecular systems were explicitly solvated with TIP3P water molecules under dodecahedron hexagon periodic boundary conditions for 15 Å buffer region. The overlapping water molecules were deleted and 0.15M KCl were added, and the systems were neutralized by adding K^+^ as counter ions. Brownian motion simulation was used to relax these systems into a local energy minimum. An ensemble (NPT) was applied to maintain the constant temperature (310 K) and pressure (1.01325 bar) of the systems, and the product simulations started with a random initial velocity. Simulated trajectories and RMSD (Root Mean Square Deviation) were then visualized and calculated using Maestro graphical interfaces.

## Results

### CKB is downregulated in human solid tumors and its lower expression correlates with worse prognosis in prostate cancer patients

**Fig. 1:**
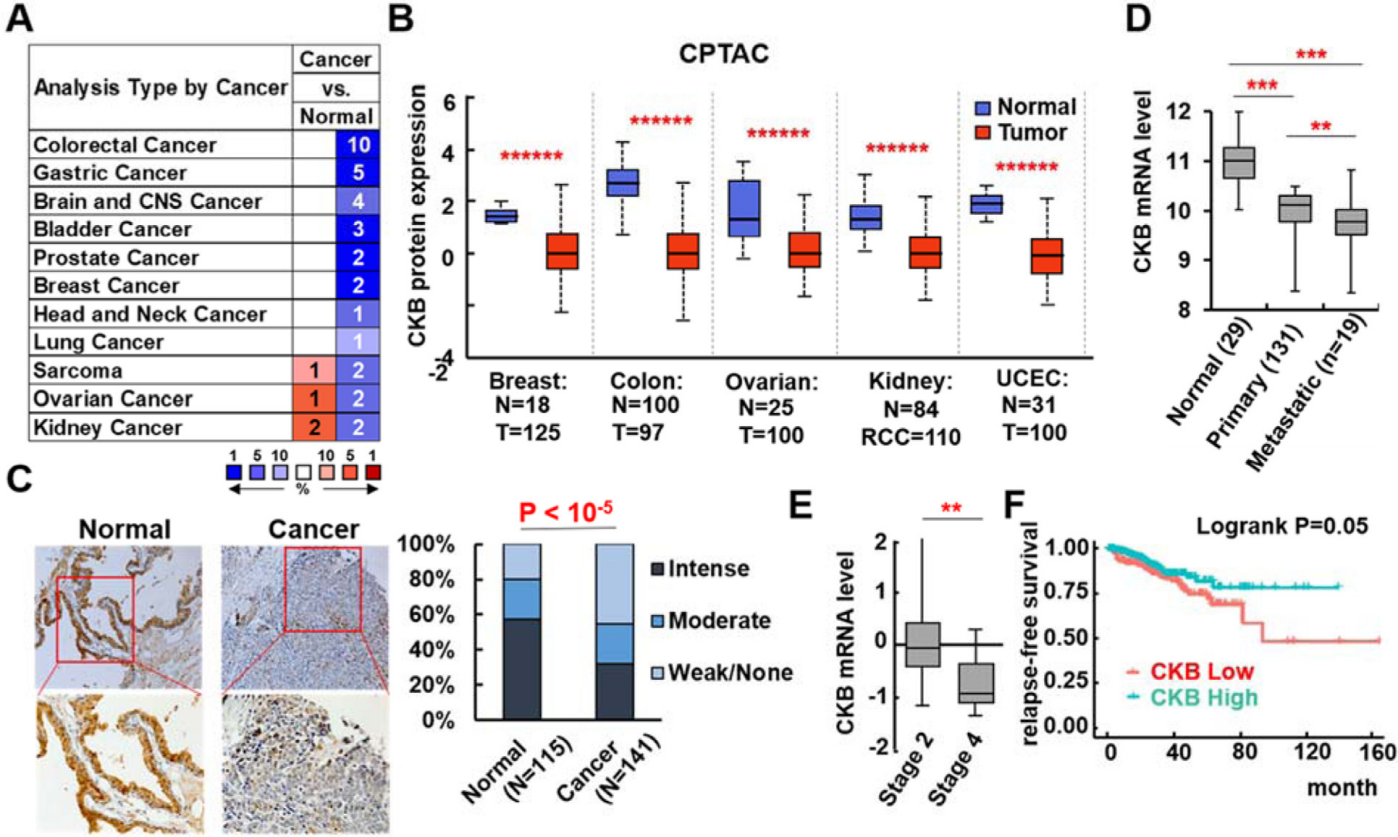
CKB is downregulated in human solid tumors, which is associated with poor prognosis. (A) CKB mRNA expression in normal vs tumor comparisons of various cancer types from Oncomine.org website. Significance thresholds are: *P* ≤ 1 × 10^−4^, fold change ≥2, and gene rank top 10%. Red signifies over-expression and blue represents under-expression in tumors. Intensities of color signify the best ranks of CKB in the analyses. The numbers represent the numbers of analyses that meet the thresholds. (B) CKB protein expression in normal and tumor samples, and the *P* values, were from the CPTAC database, obtained through UALCAN. (C) CKB protein expression was analyzed in prostate normal and tumor tissue microarray by immunohistochemistry. Staining was scored for each sample, and percentage of weak, moderate or intense CKB staining in normal and tumor samples was shown on the right. *P* value was from Chi Square Test. (D-F) CKB mRNA expression was analyzed in Taylor_Prostate dataset [45] (D), and in TCGA prostate cancer dataset for tumor stage (stage 2, n = 186 vs stage 4, n = 11) (E), and biochemical relapse (BCR) free survival time (F). **P < 0.01, ***P < 0.001 were from 2-sided *t* test. In KM survival analysis (F), patients were categorized as CKB Low or High based on the ROC curve method, and the *P* value was from Logrank test (GraphPad Prism) (color version of figure is available online).

We previously performed a human kinome cDNA screen on 500 human kinases to identify novel regulators of EMT, using luciferase assay for promoter activity of Vimentin gene, a well-established mesenchymal maker. We identified 55 potential EMT inducers, 2 of which have been reported by us (i.e., CDKL2 and ZAK) [16,17]. We also identified several potential repressors of EMT, including brain-type creatine kinase (CKB). We selected CKB for further study as potential repressor of cancer progression because its mRNA expression is significantly downregulated in many solid cancer types compared to normal controls (Fig. 1A). Its protein expression is also repressed in several solid cancer types in NCI's CPTAC cancer proteomics studies (Fig. 1B). Our analysis of CKB protein expression in human prostate normal and tumor tissue microarrays concords with this observation (Fig. 1C). We found that CKB protein levels are significantly lower in prostate cancers (n = 141) than in normal prostate tissues (n = 115) (Chi-Square=21.5, *P* < 1.0E-5).

For a detailed study on CKB, we chose to focus on prostate cancer, one of the cancer types of our interest. CKB mRNA is consistently downregulated in human prostate cancers compared to normal protein tissues across multiple well-cited datasets (Fig. 1D and Figure S1A). Moreover, a lower CKB mRNA level is significantly associated with worse prognosis in prostate cancer patients, such as higher tumor stage and grade and future relapse (Fig. 1E and S1B). Of note, patients whose prostate tumors expressing less CKB have faster biochemical relapse (rebound of serum PSA) (Fig. 1F, TCGA). Interestingly, CKB is also downregulated in mouse prostate tumors from 2 independent studies on prostate-specific *Pten* knockout mice [46], a classic prostate adenocarcinoma mouse model (Figure S1C). In line with this observation in mouse models, we also found that CKB is expressed significantly lower in TCGA patient samples where PTEN is lost (deleted and/or mutated in at least 1 copy) (Figure S1D). PTEN loss has been strongly associated with poor prognosis in prostate cancer patients (as summarized in a recent review [47]). This further supports that low expression of CKB is a poor prognosticator in prostate cancer.

Analyzing 287 human tumors that have matched mRNA levels (TCGA) and protein levels (CPTAC), we found that CKB mRNA and protein levels significantly correlate with each other (R = 0.397, *P* = 2.97E-12) (Figure S2A). Since a controversial role of CKB has been reported in colon cancer [20−22], we examined the association of CKB expression with colon cancer prognosis in its TCGA dataset (N = 640, access via cBioportal [39]). The bottom 15% of TCGA colon cancer tumors with the lowest CKB expression have significantly shorter overall survival as compared to all other cases (*P* = 0.025) (Figure S2B). Next, we analyzed large public RNA-seq datasets compiled by KMplot and found that the correlations of CKB expression with patient survival reach statistical significance in 7 solid cancer types. Of note, in 6 of the 7 cancer types, tumors with lower CKB associate with significantly shorter overall patient survival, including cervical, head-neck, kidney, ovarian, pancreatic cancer and sarcoma (Figure S2C-D). The only exception is thyroid cancer, which has remarkably good prognosis (Figure S2D). In summary, cancer genomics and proteomics data clearly indicate that CKB mRNA and protein expression are significantly downregulated in multiple solid cancer types, which is associated with poor prognosis and shorter survival, including in prostate cancer.

### CKB overexpression inhibits EMT, migration and xenograft tumor growth of prostate cancer cells

**Fig. 2:**
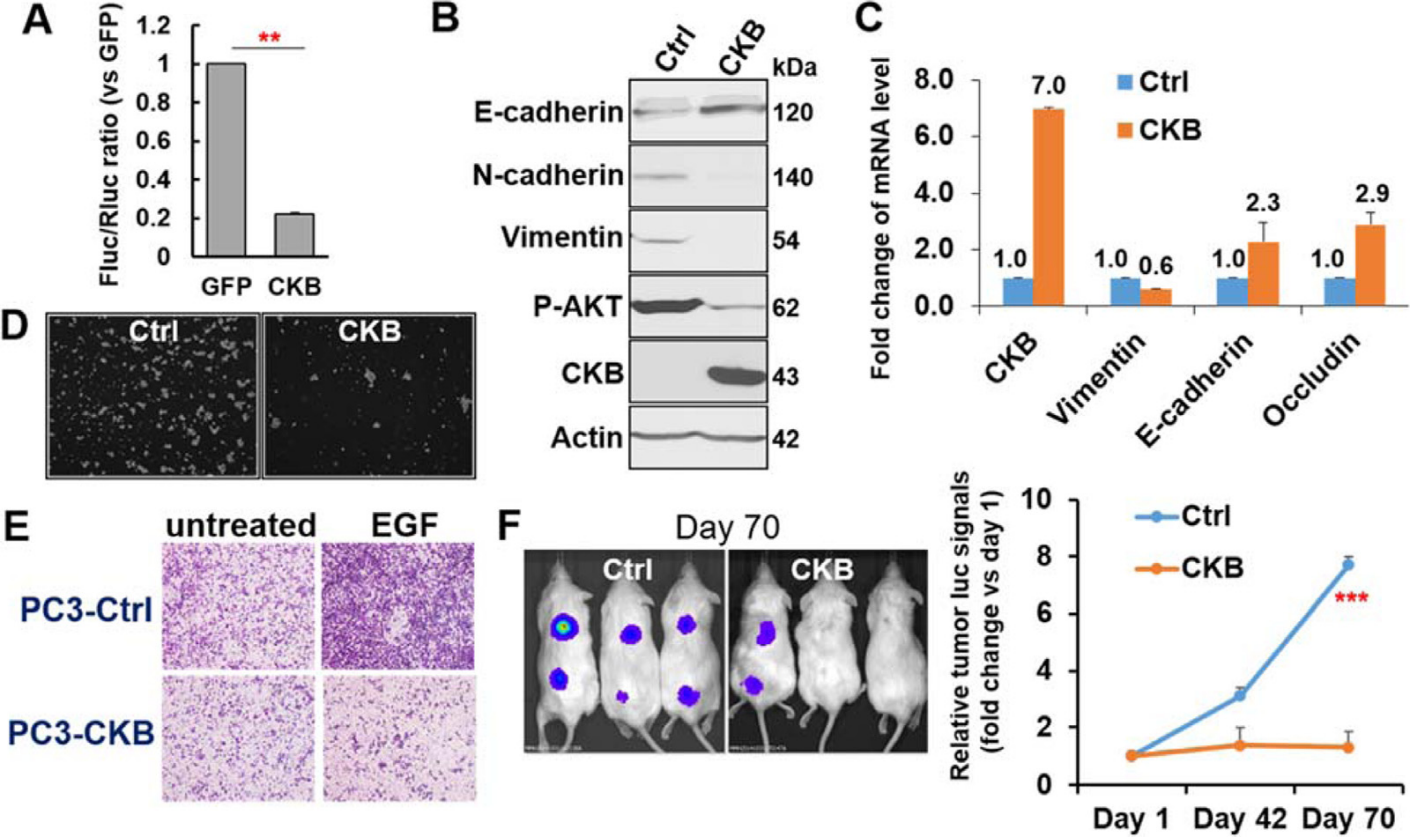
CKB overexpression inhibits EMT, migration and tumor growth of prostate cancer cells. (A) Luciferase activity in 293T cells 36 h after transfection with GFP (control, Ctrl) or CKB cDNA plasmid, together with a Vimentin promoter firefly luciferase (Fluc) reporter and a renilla luciferase (Rluc) control construct. Relative fold change of the Fluc/Rluc ratio was calculated and plotted as means  ±  SD. **P < 0.01 from triplicates. (B) Immunoblotting for indicated proteins in PC3 cells expressing GFP or CKB cDNA. (C) qPCR analysis of mRNA levels of indicated genes normalized to beta-actin in LN3 cells expressing GFP or CKB. (D) Anchorage-independent growth in a 24-well ultra-low attachment plate. (E) Cell migration as determined by Boyden chamber assay. Serum starved PC3-Ctrl and -CKB cells were treated or not treated with 20 ng/ml of EGF for 8 hr, followed by migration assays. Quantifications from duplicate experiments are in Supplementary Figure S3A. (F) 1 × 10^6^ PC3-luciferase cells expressing GFP (Ctrl) or CKB cDNA were implanted subcutaneously in male NOD/SCID mice (n = 8 or 9 mice in either group). Xenograft tumor growth was monitored by bioluminescence imaging at day 1, day 42 and 70. Fold changes relative to day 1 were calculated and plotted as means  ±  SD. ***P < 0.001 from comparing the control and CKB groups at day 70.

With evidences from our EMT cDNA screen and above-mentioned expression patterns in patient tumors, we decided to further investigate a potential role of CKB in suppressing prostate cancer progression. Consistent with results from our primary EMT screen, CKB overexpression inhibits Vimentin promoter activity (Fig. 2A). Its overexpression also induces classic epithelial markers (e.g., E-Cadherin) and reduces mesenchymal markers (e.g., Vimentin) in prostate cancer cells PC3 and LN3 (Fig. 2B−C). In line with established roles of EMT, CKB overexpression inhibits survival and proliferation of PC3 cells in low cluster plate, an anchorage-independence assay (Fig. 2D). Moreover, CKB overexpression reduces migration of PC3 cells, and ablates EGF-induced migration of PC3 cells (Fig. 2E and Figure S3A). Importantly, CKB overexpression inhibits subcutaneous (s.c.) xenograft tumor growth of PC3 cells (Fig. 2F). Comparing to day 1 after cell implantation, PC3-GFP control cell-derived xenograft tumors grew 7.7 folds, while PC3-CKB cell-derived xenograft tumors only grew 1.3 folds in 70 d (*P* < 0.001). These results on CKB ectopic expression suggest that CKB is a repressor of EMT and prostate cancer progression.

### CKB silencing promotes EMT, migration, focus formation and apoptosis resistance in vitro, as well as xenograft tumor growth and metastasis in vivo

**Fig. 3:**
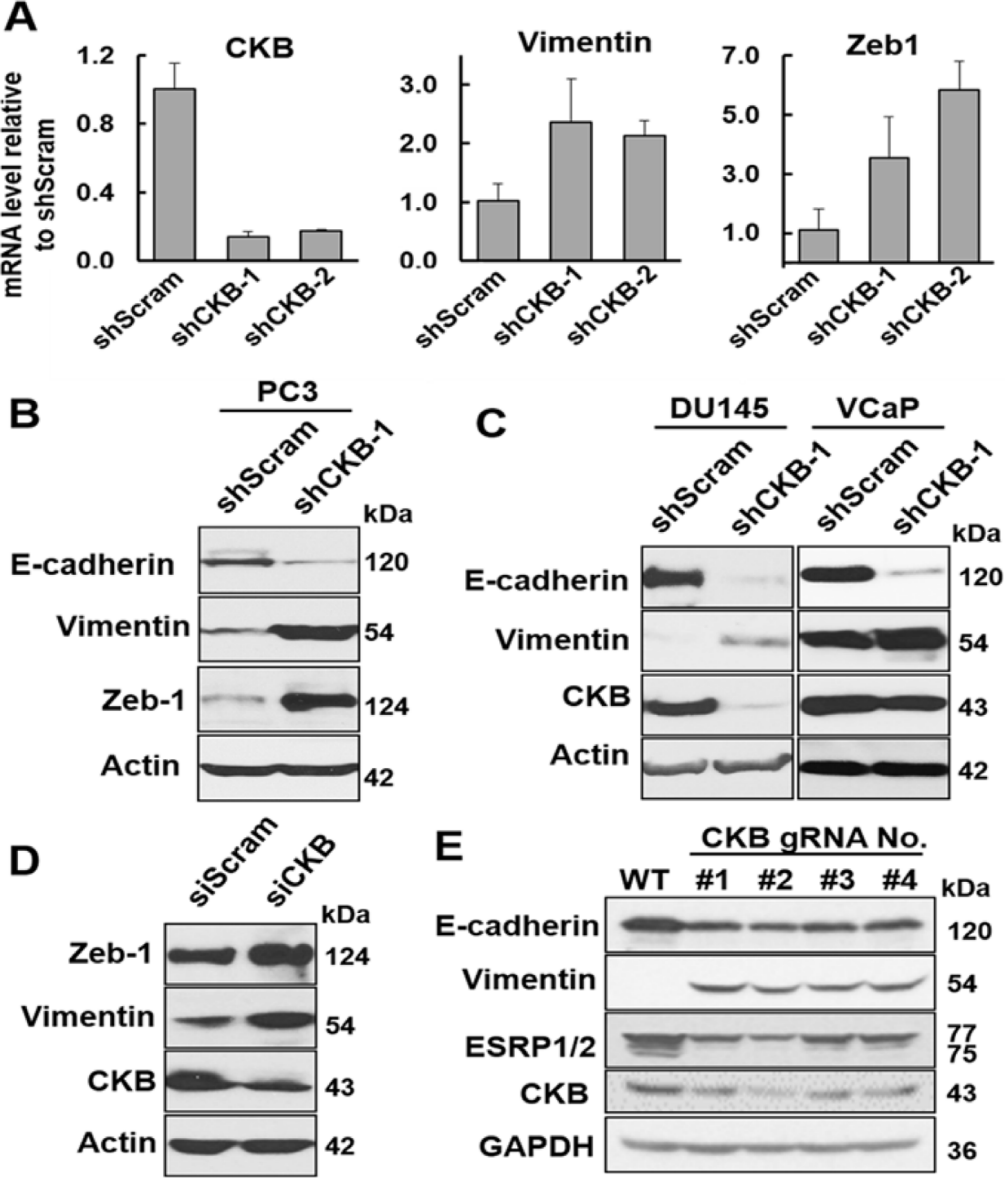
Silencing CKB induces EMT markers in prostate cancer cells. (A) qPCR analysis of PC3 cells stably expressing scramble control shRNA or 2 independent CKB shRNAs (shCKB-1 and shCKB-2). Relative fold change was calculated and plotted as means  ±  SD. *P < 0.05 comparing shScram to either shCKB group. (B) Immunoblotting of whole cell lysates of PC3 cells stably expressing scramble control shRNA or CKB shRNA. (C) Immunoblotting in DU145 and VCaP cells expressing control shScram or shCKB. (D) Immunoblotting of whole cell lysates of DU145 cells transfected with control siRNA or CKB siRNA. (E) Immunoblotting of PC3 parental cells (WT) and 4 pools of PC3 cells transfected with 4 different Cas9-gRNA plasmids targeting CKB. These PCR and immunoblotting experiments have been repeated 3 times with comparable results, and so 1 representative experiment is presented.

To recapitulate CKB downregulation as observed in patient tumors, we next investigated the impact of CKB silencing on EMT and prostate cancer progression. CKB knockdown with 2 independent shRNAs induces mesenchymal genes (Vimentin and Zeb1) in PC3 cells (Fig. 3A−B). Similarly, silencing CKB in 2 other prostate cancer cell lines DU145 and VCaP reduces E-cadherin and induces Vimentin (Fig. 3C). We obtained concordant results with siRNA-mediated CKB knockdown in DU145 cells (Fig. 3D) and CRISPR/Cas9/gRNA-mediated CKB silencing in PC3 cells (Fig. 3E). Of note, silencing CKB with 4 independent CKB gRNAs led to downregulation of E-cadherin and another epithelial marker ESRP1/2, while inducing Vimentin in PC3 cells (4 pools of cells, not single clones).

**Fig. 4:**
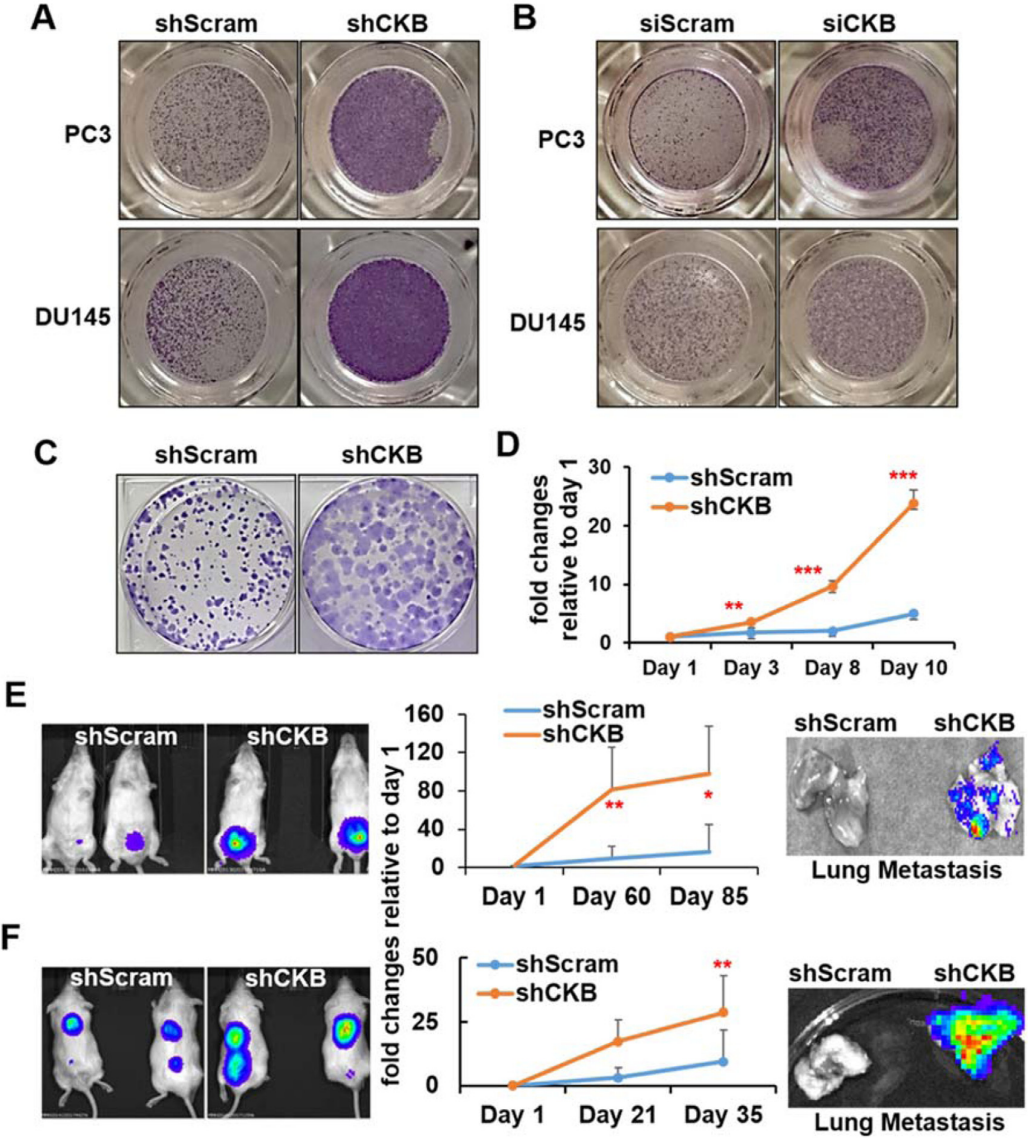
Silencing CKB promotes prostate cancer cell migration and focus formation in vitro, as well as primary tumor growth and lung metastasis in vivo. (A) Cell migration as determined by Boyden chamber assay for PC3 and DU145 cells stably expressing control or CKB shRNA. Representative pictures from triplicate experiments are shown. (B) Boyden chamber cell migration assay for PC3 and DU145 cells transfected with control siRNA or CKB siRNA. Representative pictures from triplicate experiments are shown. Quantifications are in Supplementary Figure S3B. (C) Focus formation of PC3 cells stably expressing control or CKB shRNA. Representative pictures from duplicate experiments are shown. Additional pictures are in Figure S3C. (D) Relative cell growth rate of PC3 cells expressing control shRNA (shScram) or shCKB-1 in culture medium supplied with low % of FBS (1.25%), at day 1, 3, 8 and 10 (quadruplicates). Relative fold changes to day 1 were calculated and plotted as Means  ±  SD. *P < 0.05, **P < 0.01, ***P < 0.001 from comparing shScram with shCKB-1 cells at each time points (quadruplicates). (E) PC3 cells labelled with luciferase and expressing either shScram or shCKB-1 were implanted into prostates of NOD/SCID mice (shScram n = 5, shCKB-1 n = 4 mice). Fold changes of bioluminescence readings were calculated and plotted as Means  ±  SD (middle). Representative bioluminescence images of mice before sacrifice at day 85 (left) and ex vivo images for lung metastasis after sacrifice (right) are shown. (F) DU145 cells labelled with luciferase and expressing either shScram or shCKB-1 were implanted subcutaneously in NOD/SCID mice (shScram n = 8, shCKB-1 n = 8 mice). Fold changes of bioluminescence readings were calculated and plotted as Means  ±  SD (middle). Representative bioluminescence images of mice before sacrifice at day 35 (left) and ex vivo images for lung metastasis after sacrifice (right) are shown. *P < 0.05, **P < 0.01 comparing shScram with shCKB-1 group at the indicated time points (E and F).

Phenotypically, shRNA and siRNA-mediated CKB silencing promotes migration of PC3 and DU145 cells (Fig. 4A−B and Figure S3B). CKB silencing also promotes focus formation, another assay for anchorage-independent proliferation, in PC3 cells (Fig. 4C and Figure S3C). Moreover, PC3-shCKB cells survive and grow better than PC3-control scramble shRNA (shScram) cells in culture media with reduced growth factors (1.25% FBS) (Fig. 4D). Notably, CKB silencing promotes orthotopic xenograft tumor growth and lung metastasis of PC3 cells (Fig. 4E). We obtained similar results for s.c. xenograft tumor growth of DU145 cells (Fig. 4F). In summary, CKB downregulation, as observed in patient tumors, promotes prostate tumor progression and EMT.

### AKT is phosphorylated and activated upon CKB silencing

**Fig. 5:**
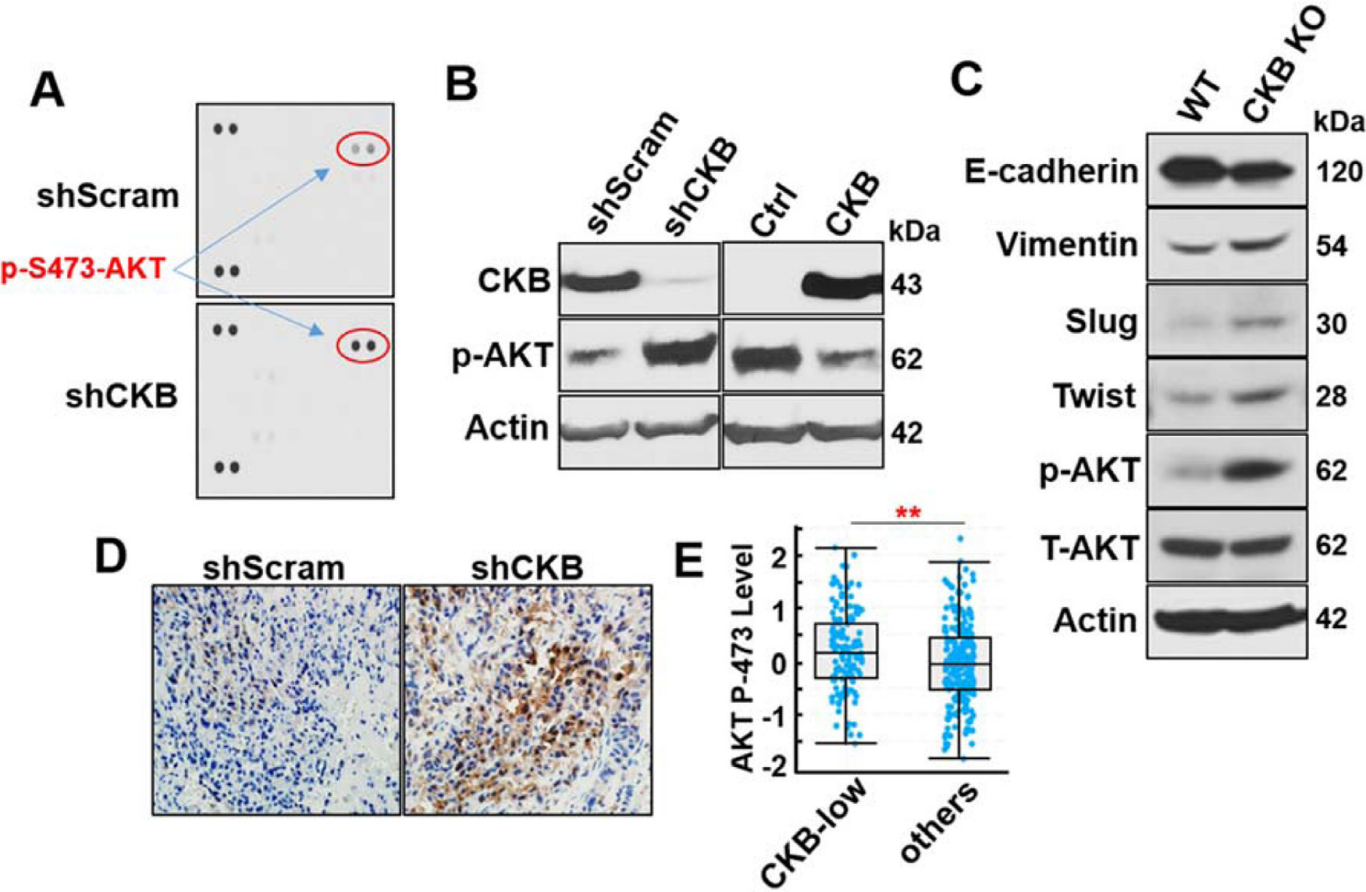
CKB silencing induces AKT-S473 phosphorylation, consistent with their correlation in patient samples. (A) Whole cell lysate from PC3 cells expressing either shScram or shCKB-1 was tested on a human phospho-kinase array (R&D Systems). Red circles show duplicate spots with signals from p-S473-AKT antibody. (B) Immunoblotting for p-S473-AKT, CKB and Beta Actin in DU145 cells expressing shScram or shCKB-1 (left), and PC3 cells carrying empty vector control (EV, Ctrl) or CKB cDNA (right). (C) Immunoblotting for E-Cadherin, Vimentin, Slug, Twist, p-S473-AKT, AKT and beta-Actin in PC3 parental (WT) and CKB knockout cells. (D) Immunohistochemistry analysis of p-S473-AKT in PC3-shScramble and PC3-shCKB xenografts. (E) p-S473-AKT levels in TCGA primary prostate tumors, as analyzed by RPPA (accessed through cBioportal), comparing samples with low CKB mRNA expression (Z-score <-0.5) with all other samples. The p-S473-AKT plot and *P* value (*P* < 0.01) were obtained from cBioPortal (color version of figure is available online).

To investigate the mechanism and downstream signaling pathways of CKB in prostate cancer cells, we performed a focused proteomics study using a Human Phospho-Kinase Array. Comparing protein lysates from PC3-shScram and PC3-shCKB cells, p-S473-AKT was the top signaling protein upregulated upon CKB silencing (Fig. 5A). p-S473-AKT induction is indicative of AKT activation, which is well established as a potent promoter for EMT, apoptosis resistance and cancer progression [[48], [49], [50], [51]]. This suggests that CKB downregulation may exert its effects by activating AKT.

To critically study the mechanisms underlying the effects of CKB silencing, besides shCKB lines, we wanted to have stable cell lines of CKB-KO. For this, we performed PC3 single cell cloning from CKB gRNA pool #2 in Fig. 3E through limited dilution in 96-well plates, followed by cell expansion. Individual clones which have DNA sequence validations of small frame shift deletions in CKB coding regions were considered as CKB-KO clones, which produced malfunctioning CKB mRNA transcripts at reduced levels. Relative to PC3 parental cells, CKB-KO and shCKB stable lines have comparable changes in mRNA levels of CKB and EMT markers (ESRP1, Vimentin, Zeb1 and Slug), showing 1 shCKB line and 1 CKB-KO clone as examples in Figure S4A.

To test our hypothesis that CKB downregulation promotes cancer progression via AKT S473 phosphorylation, we first confirmed that CKB stable silencing by shRNA and CRISPR/Cas9/gRNA increases, while its cDNA ectopic expression decreases, p-S473-AKT levels in PC3 and DU145 cells (Fig. 5B−C). Concordantly, p-S473-AKT level is also elevated in xenograft tumors from PC3 cells upon CKB silencing (Fig. 5D). Examining 352 TCGA prostate cancer patient samples that have both RNA-seq mRNA and RPPA proteomics data, we found that p-S473-AKT level is higher (*P* = 0.0032) in 36% of patient samples that have lower levels of CKB expression (Z score <-0.5) (Fig. 5E). All these results indicate that CKB downregulation activates AKT in prostate cancer cell models and patient samples.

### AKT inhibition and CKB overexpression reverse EMT induced by CKB suppression

**Fig. 6:**
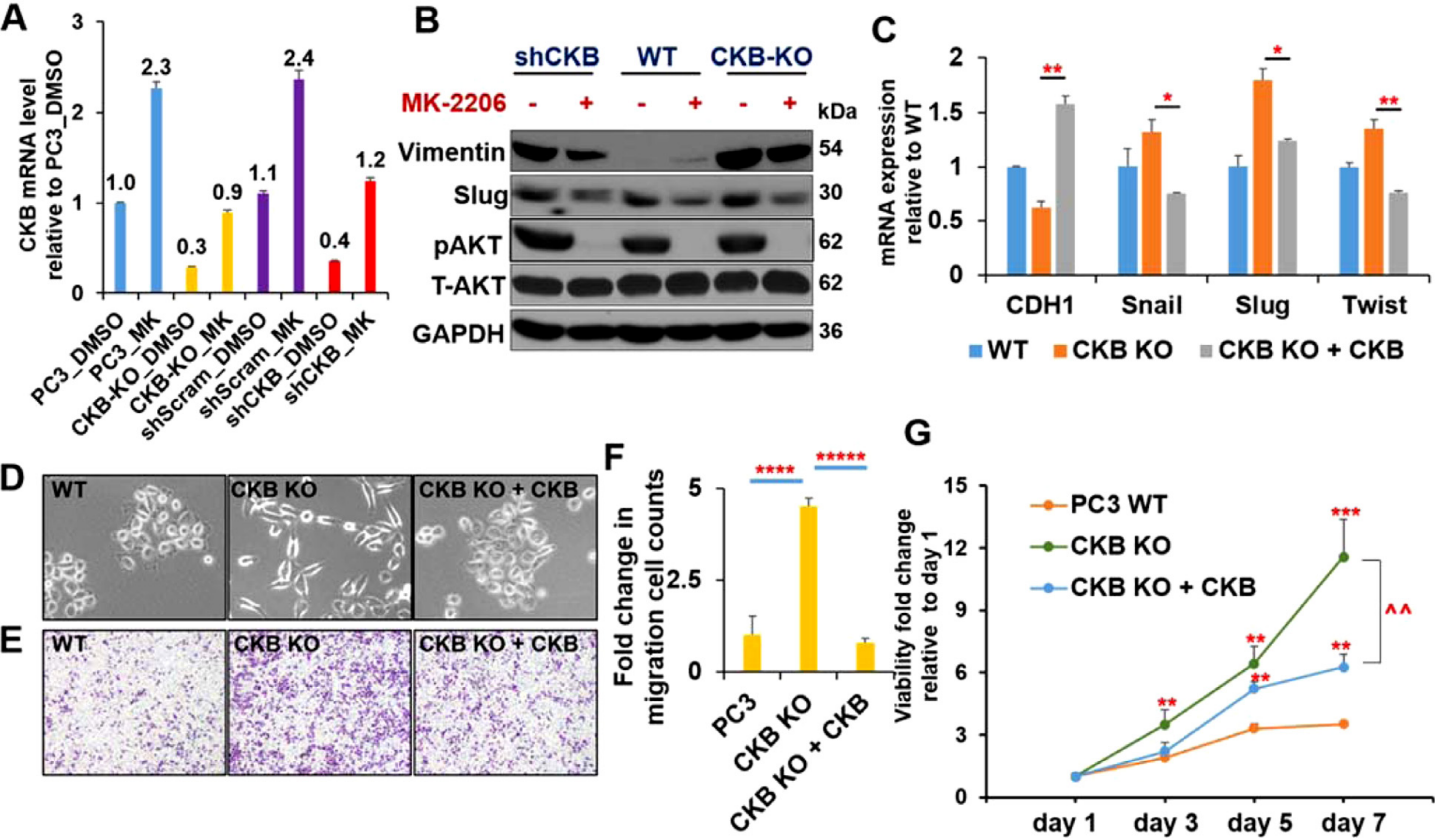
AKT inhibitor and CKB overexpression reverse EMT induced by CKB suppression. (A) RT-qPCR result of CKB expression in the indicated 4 cell lines treated with DMSO or 15uM AKT inhibitor MK-2206 for 24 h. Expression pattern for epithelial marker Occludin from these experiments is in Figure S4B. (B) Parental PC3 cells (wild type, WT), PC3 with CKB CRISPR-Cas9 knockout (CKB KO), or PC3 with CKB knockdown (shCKB-1) were treated with DMSO or 3uM of AKT inhibitor MK-2206 for 48h. Immunoblots for indicated proteins were shown. (C) qPCR analysis for indicated genes normalized to beta-Actin in PC3 parental cells (WT), CKB knockout cells (CKB KO), and CKB-knockout cells re-expressing CKB cDNA (CKB KO + CKB). *P < 0.05, **P < 0.01 comparing CKB KO to CKB KO + CKB. (D) Cell morphology were monitored by phase-contrast microscopy in PC3 WT, CKB KO and CKB KO + CKB cells. (E) Boyden chamber migration assay for PC3 WT cells, PC3 CKB KO cells and PC3 CKB KO + CKB cells. (F) Quantitative results of the migrations in E, based on triplicates. ***P < 0.001, ****P < 1 × 10^−4^, *****P < 1 × 10^−5^. (G) Alamar blue cell proliferation assay for PC3 WT, CKB KO and CKB KO + CKB cells cultured in media with reduced FBS (1.25%) at day 1, 3, 5 and 7 (quadruplicates). **P < 0.01, ***P < 0.001 comparing CKB KO cells, CKB KO + CKB cDNA cells with WT cells at corresponding time points. ^^*P* < 0.01 comparing CKB KO with CKB KO + CKB cells at day 7. These experiments have been repeated at least twice, which has yielded same conclusions. Results from a representative experiment are shown.

We next asked whether AKT activation is critical for the phenotypes induced by CKB silencing. We treated 4 PC3 lines with AKT inhibitor MK-2206 [52] and performed RT-qPCR: parental (wild type, WT) cells, CKB-KO cells, shScramble control cells and shCKB cells. Firstly, among the 4 DMSO-treated cells, we confirmed that CKB-KO and shCKB cells have comparable downregulation of CKB and Occludin, comparing to both WT and shScramble cells. Secondly, both genes were upregulated by MK-2206 treatment in all 4 cell lines (Fig. 6A and S4B). CKB upregulation by MK-2206 treatment is in line with the negative regulatory relationship between CKB and PI3K/AKT pathway.

Same conclusions could be drawn from western blotting upon MK-2206 treatments or from RT-qPCR upon treatment of another AKT inhibitor Perifosine [53]. Both AKT inhibitors [54] reversed EMT marker expression patterns induced by CKB silencing (Fig. 6B and Figure S4C). Moreover, focus (colony) formation capability of PC3-shCKB EMT cells were dramatically reduced upon 14-d treatment of MK-2206 or Perifosine (Figure S4D), further indicating that AKT activation is critical for PC3-shCKB EMT cells.

To determine whether CKB re-expression also reverses EMT phenotypes from CKB knockout, we expressed CKB cDNA in PC3-CKB-KO cells. CKB KO induces classic EMT marker expression patterns (Fig. 6C), morphological changes (Fig. 6D), cell migration (Fig. 6E and 6F), proliferation in low serum condition (Fig. 6G) in PC3 cells, all of which are reversed by CKB re-expression. These results further strengthen the link between CKB and AKT activation, and indicate that AKT activation is critical for the EMT phenotypes induced by CKB silencing.

### CKB sequestrates AKT and inhibits AKT's interaction with mTORC2 complex

**Fig. 7:**
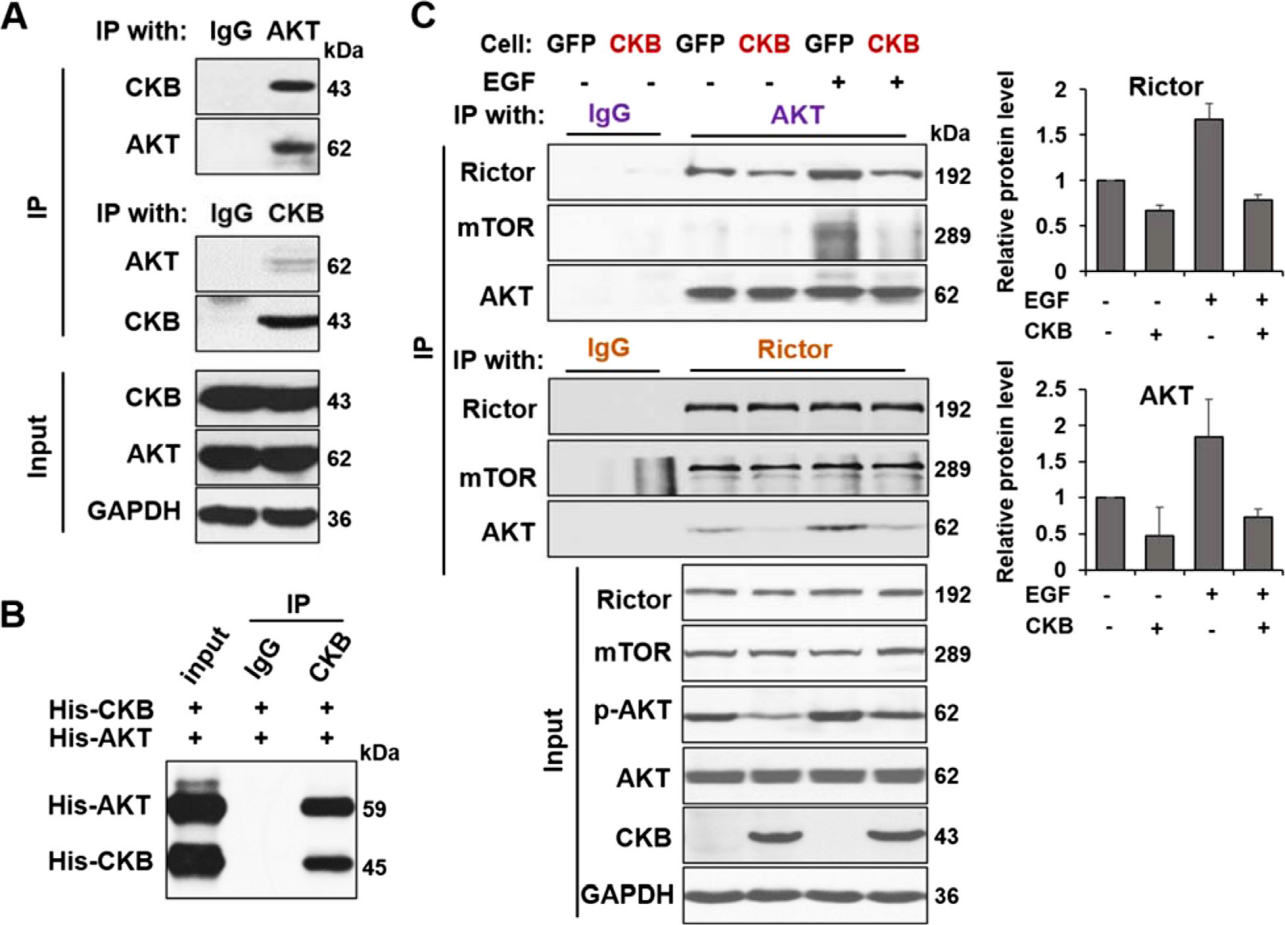
CKB interacts with AKT and inhibits AKT activation. (A) CKB and AKT proteins could reciprocally co-immunoprecipitate (co-IP) each other from LNCaP cells. (B) AKT protein was immunoprecipitated by CKB antibody in a mixture of recombinant His-tagged AKT and His-tagged CKB proteins. (C) PC3-GFP and PC3-CKB cells were treated with or without 100ng/ml EGF for 5 min. Endogenous AKT was immunoprecipated from these 2 cell lines using AKT Ab, followed by immunoblotting for Rictor, mTOR and AKT (top). Conversely, endogenous Rictor was immunoprecipated using Rictor Ab, then immunoblot for AKT, mTOR and Rictor (middle). Immunoblotting of the input whole cell lysates was shown in bottom. Fold changes of Rictor and AKT protein levels in the IP samples are plotted, relative to the sample without EGF and without CKB overexpression. The quantification is based on measurements from 2 independent experiments, using ImageJ software.

In studying the mechanism of CKB-mediated regulation of AKT-S473 phosphorylation and activation, we noticed that AKT is among the CKB interactors shown in NCBI CKB gene report. Klein et al made this initial observation in an AKT immunoprecipitation from rat CA1 hippocampal region extracts and subsequent mass spectrometry proteomics analysis [55]. We therefore speculated that CKB may inhibit AKT activation through interaction with AKT. We first confirmed that immunoprecipitation of either CKB or AKT protein from LNCaP cell extracts indeed reciprocally pulls down each other, as shown in Fig. 7A. To further test if these 2 proteins have direct physical interactions, we performed pull-down assays using purified recombinant proteins of CKB and AKT. Indeed, we found that they directly interact (Fig. 7B).

Next we set out to determine how CKB may inhibit AKT activation through interaction. S473 phosphorylation of AKT is mediated primarily by the mTORC2 complex, 1 of the 2 mTOR kinase complex. We postulated that CKB may sequestrate AKT and inhibit AKT's interaction with mTORC2 complex. To test this hypothesis, we determined whether CKB overexpression inhibits AKT's interaction with Rictor, a specific subunit in mTORC2 complex, during AKT activation. EGF is known to activate AKT in many epithelial cancer types, including prostate cancer [56]. As shown in Fig. 7C, we subjected cell lysates from EGF-treated PC3-GFP or PC3-CKB cells to reciprocal immunoprecipitation and immunoblotting of AKT and Rictor proteins. As expected, in both experiments, the AKT-Rictor interaction is elevated upon EGF treatment. Importantly, we observed a lower AKT-Rictor interaction in PC3-CKB cells than in PC3-GFP cells. This result supports the notion that CKB sequestrates AKT to reduce AKT's interaction with mTORC2 complex, thus inhibiting AKT phosphorylation.

### C-terminus of CKB interacts with PH domain of AKT, inhibits AKT activation and cell proliferation

**Fig. 8:**
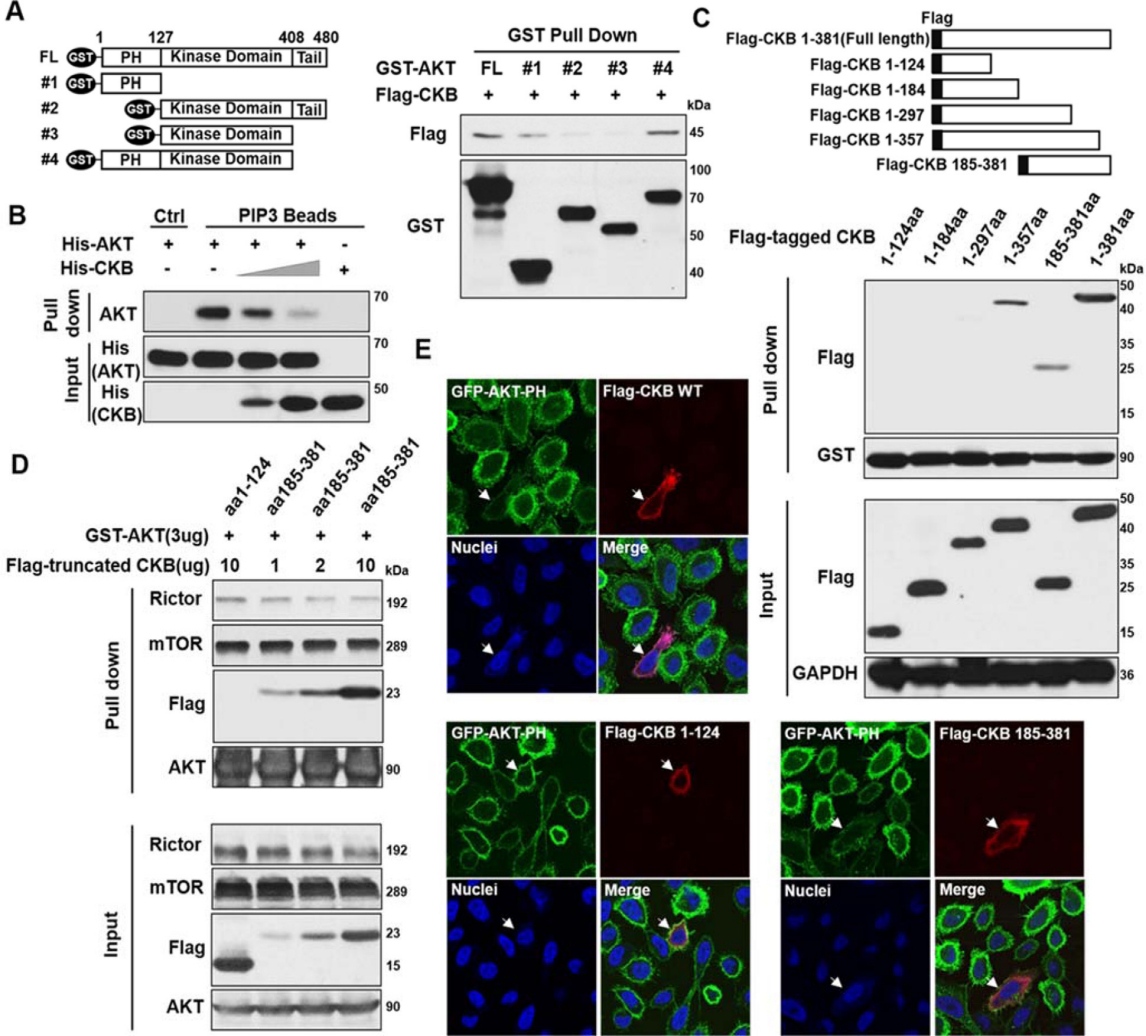
CKB interacts with AKT PH domain through its C-terminal. (A) Schematic of GST-tagged AKT full-length (FL) and truncation mutants (left). The 293T cells were transfected with cDNA vectors for GST-tagged AKT FL or truncations, together cDNA plasmid for Flag-tagged CKB FL protein. GST-tagged AKT proteins were immunoprecipitated by glutathione sepharose beads from co-transfected cells, followed by immunoblotting for Flag and GST (right). (B) PIP3 coated agarose beads were incubated with purified His-tagged AKT (2ug) and/or His-tagged CKB (0, 1 or 2ug) as indicated. PIP3 binding proteins were pulled down. Immunoblots for AKT and CKB were shown. (C) Schematic of Flag-tagged CKB FL and truncation mutants (top). GST-tagged AKT proteins were pull down by glutathione sepharose beads from 293T cells co-transfected with plasmids for GST-tagged AKT FL and Flag-tagged CKB FL or truncations as indicated (middle). Immunoblots on input whole cell lysates are in the bottom. (D) 293T cells were co-transfected with GST-tagged AKT vector and indicated amounts of Flag-tagged CKB truncation vectors. GST-tagged AKT proteins were pull down by glutathione sepharose beads from these cells. Immunoblots for Rictor, mTOR, Flag and AKT were analyzed. (E) Representative immunofluorescence images for GFP-AKT-PH fusion protein (green), Flag-CKB FL protein or Flag-CKB truncations (red) and nuclei (blue). PC3 cells expressing GFP-AKT-PH were transfected with Flag-CKB plasmids. White arrows indicate the PC3 cells transfected with the corresponding CKB full length or truncated cDNA constructs. Untransfected cells in the same wells serve as controls. Additional representative images are in Supplementary Figure S5. Quantifications of GFP-AKT-PH signal ratios on membrane vs cytoplasm in multiple untransfected and transfected cells are presented in Figure S6A. These immunoblotting and IF experiments have been repeated twice, which has yielded same conclusions. Results from a representative experiment are shown (color version of figure is available online).

To further understand the detailed mechanisms of CKB in regulating AKT activation, we next set out to determine the interacting domains between AKT and CKB. Firstly, full length CKB cDNA with Flag tag was co-transfected with full length or 4 truncated AKT cDNAs with GST tag [34] in 293T cells. Interestingly, as shown in Fig. 8A, CKB protein mainly interacts with the PH domain of AKT, a critical domain of AKT phosphorylation and activation. Upon stimulation, AKT is recruited to membrane through the binding of its PH domain to the PIP3 that is generated by PI3K [24,57]. To examine the relationship of PIP3, AKT, and CKB proteins, we performed an in vitro competition assay incubating PIP3-beads with recombinant AKT proteins in the presence of different amounts of purified CKB protein. As shown in Fig. 8B, interaction of AKT protein with PIP3 decreases as the amount of CKB protein increases. These results indicate that CKB interacts with the PH domain of AKT, and CKB competes with PIP3 for the binding of AKT protein.

Secondly, we set out to determine which CKB domain(s) interacts with AKT protein. We generated 5 CKB cDNA truncations as previously described [58,59]. Plasmids for full length and 5 truncated CKB cDNAs with Flag tag were co-transfected with plasmids for full length AKT cDNA with GST tag into 293T cells. GST pull down assay showed that aa185-381, the C-terminal part of CKB protein, interacts with AKT (Fig. 8C). We further found that the AKT-Rictor interaction decreases as the level of this CKB C-terminal domain (aa185-381) increases in a dose-dependent fashion, while the N-terminal domain of CKB protein (aa1-124) does not immuno-precipitate with AKT (Fig. 8D).

To further confirm our findings, a PC3 cell line stably expressing a fusion protein of GFP with PH domain of AKT (GFP-AKT-PH) were transfected with various Flag-CKB constructs, followed by EGF treatment and then immunofluorescence staining of Flag and GFP signals. Consistent with the results shown thus far, PC3 cells manifest less GFP-AKT-PH membrane distribution when cells were co-expressed with full length or aa185-381 CKB constructs (Fig. 8E, S5 and quantifications in Figure S6A). In contrast, expressing the N-terminus of CKB protein (aa1-124) failed to inhibit signal and membrane localization of GFP-AKT-PH in PC3 cells (Fig. 8E). In summary, these results indicate that the C-terminal domain of CKB protein is the main domain responsible for interacting with AKT and inhibiting membrane trans-localization of AKT-PH domain.

**Fig. 9:**
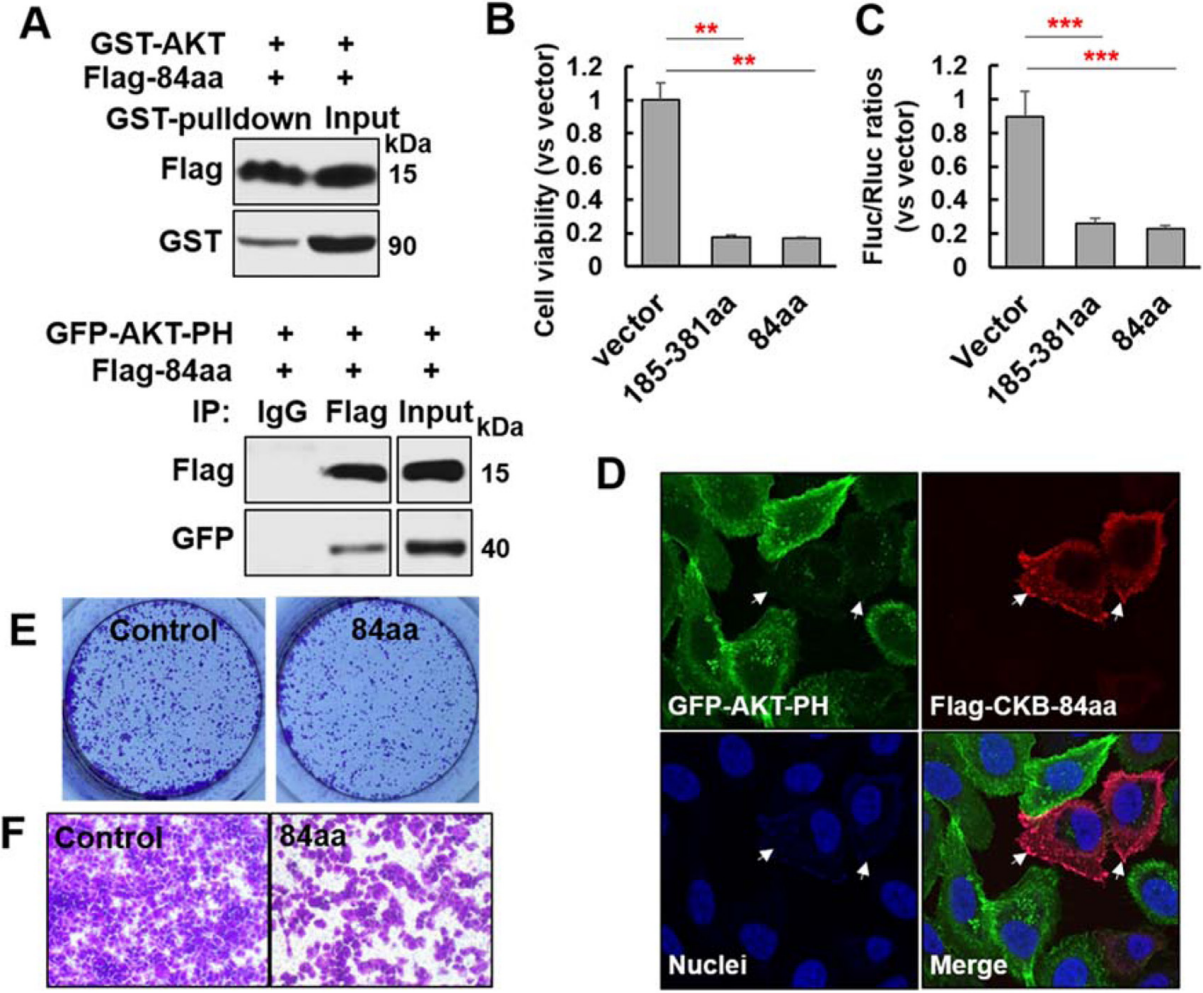
C-terminal 84aa fragment of CKB protein inhibits Vimentin promoter activity, focus formation and migration of PC3 cells. (A) (Top) GST-tagged AKT proteins were pull down by glutathione sepharose beads from 293T cells co-transfected with plasmids for GST-tagged AKT FL and Flag-tagged CKB-84aa (298aa-381aa), as indicated. Immunoblots for Flag and GST were shown. (Bottom) Immunoprecipitation assay using anti-Flag antibody from 293T cells co-transfected with plasmids for GFP-AKT-PH and Flag-tagged CKB C-terminal 84aa fragment. Immunoblots for Flag and GFP were shown. (B) Cell proliferation assay measured by alamar blue in 293T cells transfected with Flag empty vector, Flag-tagged CKB-185aa-381aa or Flag-tagged CKB-298aa-381aa (84aa) plasmids. (C) Luciferase activity in lysates co-transfected with Flag vector, Flag-tagged CKB-185-381aa or Flag-tagged CKB-84aa plasmids, together with Vimentin promoter firefly luciferase reporter and pGL4.74 renilla luciferase plasmids in 293T cells. Twenty-four hours later, cells were lysed for measuring luciferase activity. **P < 0.01, ***P < 0.001 from 2-sided *t* test comparing either CKB construct to vector control (triplicates). (D) Representative immunofluorescence images for GFP-AKT-PH (green), Flag-CKB 84aa fragment (red) and nuclei (blue). PC3 cells expressingGFP-AKT-PH were transfected with plasmid for Flag-CKB-84aa. White arrows indicate the PC3 cells transfected with Flag-CKB-84aa construct. Untransfected cells in the same wells serve as controls. Quantifications of GFP-AKT-PH signal ratios of membrane vs cytoplasm in untransfected (not red) and transfected (red) cells are presented in Figure S6B. (E) Focus formation assay of PC3 cells infected with lentivirus carrying either vector control or CKB-84aa. (F) Cell migration determined by Boyden chamber assay in PC3 cells infected with lentivirus carrying either vector control or CKB-84aa. Quantifications of focus formation and migrations (triplicates) are in Figure S6C-D. These experiments have been repeated at least twice, which has yielded same conclusions. Results from a representative experiment are shown (color version of figure is available online).

Since the aa1-297 of CKB protein does not, while aa185-381 does, interact with AKT (Fig. 8C), we inferred that the C-terminal 84 amino acid of CKB protein (aa298-381) is the key CKB domain interacting with AKT. To validate this reasoning and to test if this C-terminal fragment interacts with AKT's PH domain, we co-transfected a cDNA construct for CKB aa298-381 (84aa) fragment with cDNA construct for either AKT full length or its PH domain. As shown in Fig. 9A, the C-terminal 84aa fragment of CKB indeed interacts with AKT full-length protein and its PH domain. Given the crucial role of AKT signaling in survival and proliferation of prostate cancer cells, we next set out to test if the 84aa CKB fragment inhibits EMT, survival and proliferation of prostate cancer cells. Indeed, CKB-84aa inhibits Vimentin promoter activity and cell viability (Fig. 9B−C), manifesting similar phenotypes as C-terminal part of CKB (aa185-381). Of note, CKB-84aa also reduces the membrane distribution pattern of AKT-PH domain upon EGF treatment (Fig. 9D, quantifications in Figure S6B), as well as reduces focus formation and migration of PC3 cells (Fig. 9E−F and quantifications in Figure S6C-D). These results indicate that the C-terminal 84aa fragment of CKB is crucial for CKB's regulation of AKT activation, EMT and cancer cell proliferation.

### AKT's PH domain and CKB's C-terminal 84aa fragment establish their major interaction interface in structure-based co-evolution analysis and molecular dynamics simulations

**Fig. 10:**
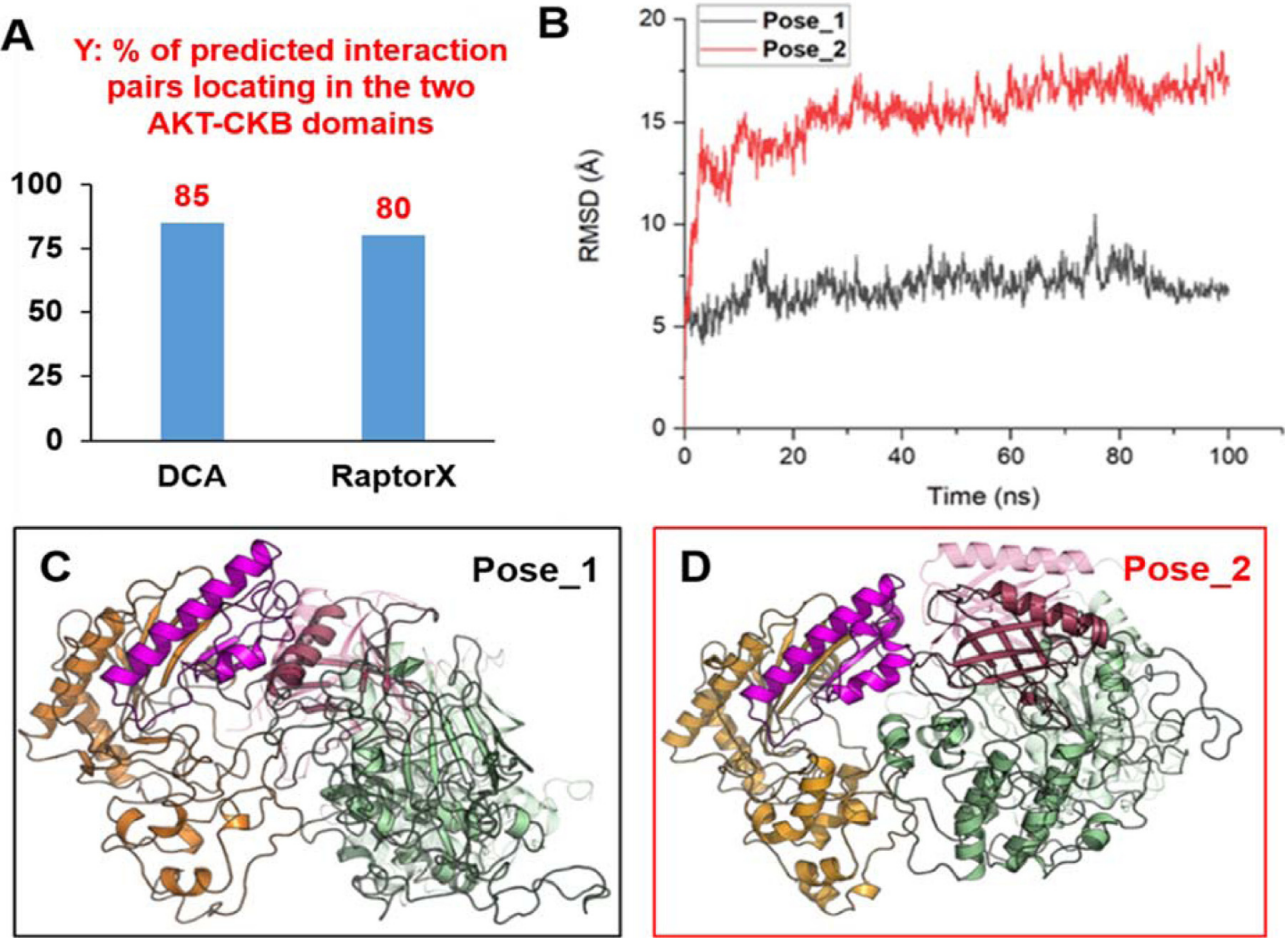
AKT's PH domain and CKB's C-terminal 84aa fragment establish the major interaction interface of these 2 proteins in molecular dynamics simulations. (A) Potential interaction residues between AKT and CKB proteins were predicted using 2 different co-evolution techniques DCA and RaptorX, based on their amino acid sequences. Y axis: Percentage (%) of the top 20 predicted interaction amino acid pairs that reside in the PH domain of AKT and the C-terminal 84aa fragment of CKB. The top 20 residue pairs were listed in Supplementary Figure S3A. (B) The RMSD of Cα atoms of AKT and CKB in 100 ns trajectory. RMSD (Root Mean Square Deviation) means that the structural variability between the structures with the initial structure in molecular dynamics simulation. A higher RMSD value means grater differences with initial structure. (C) and (D) The initial and final structures for the 2 binding modes before and after molecular dynamics simulations. The initial structure of AKT before molecular dynamics simulation is shown as transparent mode. The Pose_1 indicates the simulation results for binding pose illustrated in Fig. 10C and the Pose_2 is for Fig. 10D.

Since crystal structures of AKT and CKB were available, we decided to independently investigate their interaction using computational approaches. Firstly, we started with less resource-costly methods based on co-evolution theory. Co-evolution theory was originally conceived to assist in predicting protein structure by inferring covariant pairs of interacting residues [60] and later extended to identify hot spot residues on the interface of protein-protein interactions [61]. We used 2 well-known co-evolutional techniques, DCA (Direct Coupling Analysis) [60] and RaptorX [62], which were based on different algorithms. They were used to uncover possible critical residues involved in AKT-CKB interaction. Top 20 pairs of potential interaction residues ranked by either method were listed in Figure S7A. Interestingly, the majority of top 20 ranked pairs belong to the 84aa fragment of CKB and the PH domain of AKT, i.e., 17 pairs from DCA and 16 pairs from RaptorX analysis (Fig. 10A). Our experimental results indicate that CKB-AKT interaction was between the 84aa fragment of CKB (aa298-381) and the PH domain of AKT (aa1-110) (Figs. 8−9). It is encouraging that the prediction results of the 2 co-evolution methods highly agreed with each other, and were in line with our experimental results. This indicate that the predicted results from co-evolutional techniques were relatively reliable.

Secondly, in order to further view their interaction on crystal structures, we performed protein-protein docking simulations. Crystal structures of AKT and CKB were obtained from PDB [63,64]. We randomly selected 2 pairs of residues from co-evolution analysis as the input information of active binding sites for the docking simulations. Three candidate binding modes were obtained, as illustrated in Figure S7B-S7D. Clearly, the predict binding pose in Figure S7D did not agree with the co-evolutionary results in Fig. 10A and S7A, with no contacts between PH domain of AKT and the 84aa fragment of CKB. Hence, we further evaluated the binding poses in Figure S7B-C via more resource-costly molecular simulation methods.

Finally, to determine which binding mode in Figure S7B and S7C was more stable, we performed large time scales of molecular dynamics simulations to relax and adjust the conformations. According to the fluctuation trend of Root Mean Square Deviation (RMSD, indicative of structural variability) for the complex of AKT-CKB along the trajectory, the complex reached a stable state after 30 ns of simulations (as shown in Fig. 10B) and much longer simulations were extended to 100ns. The final complex structures were obtained and presented in Fig. 10C−10D. The Pose_1 for the binding pose illustrated in Fig. 10C has lower RMSD and was considered more stable than the Pose_2 for Fig. 10D. The Pose_1 stayed fairly stable, where it was evident that AKT's PH domain and CKB's 84aa C-terminal fragment established their major interaction interface. In contrast, the AKT structure in the Pose_2 has undergone obvious conformational change (higher RMSD), especially the PH domain (aa1-110), which indicated that the binding Pose_2 no longer agreed with the co-evolutionary results. Therefore, our structure-based computational analyses have independently validated the AKT-CKB interaction domains that we observed using experimental approaches.

In summary, we have uncovered that brain-subtype creatine kinase (CKB) is a crucial suppressor of EMT and prostate cancer progression through inhibiting AKT activation. CKB is consistently downregulated in several solid cancer types and its lower expression significantly correlates with worse prognosis. Its overexpression represses, while its silencing induces, AKT activation, EMT, cell migration and xenograft tumor growth of prostate cancer cells. Mechanistically, the C-terminal fragment of CKB physically interacts with the PH domain of AKT, which sequestrates AKT and competes with PIP3 and mTORC2 for AKT binding. Importantly, we have identified a new mode of negative regulation in AKT activation.

## Discussion

EMT has been strongly implicated in invasion, metastasis and drug resistance of tumor cells. A majority of studies and existing knowledge on EMT regulation is about EMT transcriptional regulation involving EMT-TFs [65]. Negative regulators of EMT should also be crucial in controlling EMT, which is far less characterized. In this study, we elucidate a novel EMT suppressing kinase, CKB, and its new mode of action. We found that CKB inhibits EMT through post-translationally blocking the activation of AKT that is a critical event for EMT.

CKB's role in colon cancer has been reported. In an earlier study, Mooney et al reported that overexpressing the kinase dead version of CKB cDNA (i.e., C283S mutant) promoted EMT, while overexpressing its wild type cDNA protected colon cancer cells from stress, such as glucose deprivation and heat [21]. Two more recent studies drawn different conclusions in CKB's role in liver metastasis of colon cancer cells, both starting with CKB being a target of some miRNAs in colon cancer cells. Loo et al showed that CKB is a target of 2 metastasis-inhibiting miRNAs miR-551a and miR-483. Very intriguingly, Loo et al showed that CKB is released into the extracellular space by metastatic colon cancer cells to utilize the abundant creatine in liver tissue to produce phosphocreatine, which is imported into cancer cells to generate ATP [20]. As pointed out by Loo et al [20], CKB's capability in harnessing creatine abundance in liver tissue may be key for its role in promoting liver metastasis of colon cancer cells in their studies. In another study, Torres and colleagues demonstrated that CKB is 1 of the 2 shared targets of 3 metastasis-promoting miRNAs (miR-424-3p, -503, and -1292), and CKB downregulation increased cell adhesion and proliferation in colorectal cancer cells, as well as their seeding in liver in vivo [66]. Torres et al also showed that lower levels of CKB protein are significantly associated with shorter survival in colorectal cancer patients.

The reason that we studied CKB as a top candidate for EMT negative regulators identified from our cDNA screen is that its downregulation consistently correlates with poor prognosis in several cancers (Figure S2B-C). These are in line with the putative roles of EMT in cancer development and progression [67]. CKB's role in cancer types other than colon cancer is largely unknown, especially in those cancers where liver is not the dominant metastatic site, such as prostate cancer. By characterizing EMT marker expression and cellular phenotypes, we have validated that CKB overexpression inhibits, while its silencing promotes EMT and prostate cancer growth. Subsequent studies are warranted to determine whether CKB has a similar role and mechanism in other caner types where its lower expression is also associated with shorter survival (Figure S2B-C).

We further strengthened our conclusions by performing CKB cDNA rescue experiment in PC-CKB-KO cells. CKB re-expression reversed EMT gene expression patterns, cell morphology, migration and proliferation induced by CKB KO (Fig. 6). Note that CKB re-expression significantly reversed, but did not fully rescue, the effects of CKB KO on PC3 cell proliferation under reduced serum condition (Fig. 6G). This may be because CKB function is highly compartmented in cells [68] and a substantial portion of exogenous CKB protein may not be compartmented properly to fully recover the loss of endogenous CKB function.

Mechanistically, we found that inhibiting AKT activation is a critical part of CKB's mechanism of action in prostate cancer progression. AKT family of protein kinases plays a key role in insulin signaling, cellular survival, and transformation [24,25]. AKT activation relies on a balance of positive and negative regulation [24]**.** Positive regulation of AKT activation is well established, such as RTK, PI3K, mTOR signaling [24] and many others [9−12,69]. Conversely, negative regulation of AKT signaling is less characterized, where a few phosphatases have been implicated, including PTEN, PP2A, PHLPP, and INPP4B [[70], [71], [72]]. Our study reveals a novel mode of negative regulation of AKT activation. We demonstrated that CKB inhibits AKT activation by physically interacting with AKT and sequestrating AKT from its interaction with PIP3, which reduces AKT's membrane translocalization and activation.

AKT activation has been shown to induce EMT through regulating EMT-promoting transcription factors, such as Slug, Snail and Twist, thus inhibiting E-cadherin expression [49,73]. Our study has revealed CKB as a new AKT interactor and negative regulator in EMT. It is conceivable that CKB inhibits EMT through regulating AKT and its downstream EMT-promoting transcriptional factors. As AKT activation is crucial for many cancer types, CKB downregulation, as seen in multiple epithelial cancer types, may be an integral part of general mechanisms underlying AKT activation beyond prostate cancer, which warrants further investigation. Furthermore, it is worth investigating whether CKB's sequestration of AKT activation occurs in other biological processes and pathological conditions where AKT activation has been shown to be critical [24,25].

CKB and another cytosolic creatine kinase CKM have been shown to co-localize with E-cadherin at apical adherence junctions of intestinal epithelial cells, where they are critical for junction assembly and epithelial integrity [74]. In colitis mouse models induced by dextran sulfate sodium or 2,4,6-trinitrobenzene sulfonic acid, expression of CKB is reduced, and intestinal epithelial integrity is compromised with increased intestinal permeability [74]. Concordantly, in inflammatory bowel disease patient tissues, CKB expression is significantly downregulated as compared to tissues from healthy control individuals. Given that CKB is suggested to be critical for epithelial integrity [74], downregulation of CKB may be a part of general mechanisms underlying epithelial cell's loss of their polarity and integrity during the process of tumor transformation.

CKB has been shown to be downregulated by miRNAs in colon cancer [20,66,75] or by promoter methylation in gastric cancer [23]. It is worth investigating how CKB is downregulated in many epithelial cancers, which may lead to finding novel agents to induce CKB expression as a potential therapeutic strategy. Interestingly, CKB is downregulated in prostate tumors from prostate-specific *Pten* conditional knockout models in at least 2 independent studies (Figure S1C). Since *Pten* knockout activates PI3K/AKT pathway, this suggests that CKB expression may be downregulated by AKT activation, as a negative feedback regulation. Supporting this hypothesis, Garcia et al reported an interesting observation that blood creatine kinase level is elevated in patients treating with inhibitors for EGFR, PI3K or MEK. Since they could not distinguish creatine kinase isoforms from the blood tests, they further studied in human keratinocytes in culture, where they found that CKB isoform is induced by the inhibitors, but not CKM or mitochondrial creatine kinases [76]. We also found that AKT inhibitor MK-2206 induces CKB mRNA expression in PC3 cells (Fig. 6A). These observations further support that AKT activation may, at least in part, be responsible for CKB's downregulation in cancer, which also warrants further investigations.

Finally, through mutation analysis, we have mapped the CKB-AKT interaction domains on both proteins. We have further narrowed down the critical interaction domain on CKB to be its C-terminal 84aa fragment (aa298-318). This fragment reduces Vimentin promoter luciferase activity, membrane localization and intensity of AKT-PH domain upon EGF stimulation, focus formation and migration of prostate cancer cells (Figs. 8-9). These results suggest that this fragment has a potential to become a therapeutic agent, if peptides derived from its smaller fragments exert similar activities. This may become an attractive alternative approach to block PI3K/AKT pathway. Interestingly, we independently identified these interaction domains using structure-based computational approaches (Fig. 10), which further supports that the CKB-AKT relation that we have identified may exist in other biological contexts, given that both proteins are ubiquitously expressed in a number of cell types.

Overall, our study indicates that CKB downregulation is associated with poor prognosis in prostate cancer, and CKB downregulation functionally promotes prostate cancer progression, at least in part by promoting AKT activation and EMT. CKB acts by sequestrating AKT and blocking AKT's activation by mTOR kinase. We found that the C-terminal 84aa of CKB interacts with AKT's PH domain, which is key for CKB's action. Therefore, CKB's underexpression may be a poor prognosticator and its C-terminal fragment may become a starting point of developing peptide therapeutics for prostate and other cancer types.

## Acknowledgments

We are grateful to Dr. Keqiang Ye and Dr. Christine Gilles for providing us the AKT cDNA constructs and Vimentin promoter luciferase construct, respectively. We also would like to thank other members of the Li lab for discussions and assistance. We apologize to the colleagues whose works were not cited due to space constraint.

## Author contributions

W. Li, Z. Wang, and M. Hulsurkar conceived and designed the projects. Z. Wang, M. Hulsurkar, L. Zhuo, J. Xu, H. Yang, S. Naderinezhad, L. Wang, G. Zhang, N. Ai, L. Li, J. Chang, L. Fazli, C. Creighton and F. Bai performed experiments and/or carried out data analysis. M. Ittmann provided human prostate tissue microarray. M. Ittmann, Z. Wang and S. Zhang examined IHC staining results. M. Gleave provided technical assistance and helpful discussion. W. Li and Z. Wang wrote the manuscript with inputs from other authors. W. Li supervised the project.

## Supplementary Info

Supplementary Figure S1. CKB is downregulated in prostate tumors in patients and mouse models. (A) CKB mRNA expression are analyzed in normal adjacent prostates (n=63), prostate primary tumors (n=65) and metastatic tumors (n=25) in Yu dataset (GSE6919). (B) CKB mRNA expression are analyzed in prostate cancer patient samples with (n=38) or without (n=42) future relapse (Glinsky dataset), or with different Gleason grades in TCGA dataset (Gleason score 6-7, n=291 vs Gleason score 8-10, n=207) (TCGA data through cBioPortal in July 2020). (C) Mouse CKB mRNA levels are analyzed in two PTEN knockout prostate cancer datasets as indicated. (D) CKB expression in PTEN-WT vs PTEN-loss patient tumors in the TCGA prostate cancer dataset (downloaded from cBioportal). PTEN mutations or loss of one or both alleles are considered PTEN-loss. *P<0.05, **P<0.01, ***P<0.001, from Student t-test.

Supplementary Figure S2. Patients with lower CKB expression correlate with poor overall survival. (A) CKB mRNA-protein correlation in 287 human tumors that have both CKB mRNA levels (TCGA) and protein levels (CPTAC) (R=0.397, P=2.97E-12). (B) Kaplan-Meier curves comparing TCGA colon cancer patients with altered CKB expression (15% samples with lower CKB expression, Z score < -0.65) vs all other patients (accessed through cBioPortal in July 2020). (C-I) Kaplan-Meier curves were shown for 7 solid tumor types where the Logrank P values were <0.05 for the association of CKB expression with survival of cancer patients (either positively or negatively). Six of seven cancer types show a negative correlation. CKB expression was defined as high or low in each RNA-seq datasets based on the best split. Plots and statistic data were downloaded from KMPlot (http://kmplot.com/analysis/index.php?p=service&cancer=pancancer_rnaseq).

Supplementary Figure S3. CKB overexpression ablates EGF-induced migration in PC3 cells, while its silencing promotes migration and colony formation. (A) Quantitative data from image analysis using Image J software on the migration experiments (triplicates) in Figure 2E. (B) Quantitative data from image analysis using Image J software on the migration experiments (triplicates) in Figure 4B. (C) Images of focus formation of PC3-shScram and shCKB cells in 6-well plates (duplicates). The sparsely seeded cells were grown for 12 or 14 days with fresh media replenishment every 5-6 days, followed by fixation and crystal violet staining.

Supplementary Figure S4. AKT inhibitors MK-2206 and Perifosine inhibit EMT gene expression and focus formation of PC3 CKB-KO and shCKB cells. (A) Assessed by RT-qPCR, CKB and epithelial gene ESRP1 are lower, while mesenchymal genes Vimentin, Zeb1 and Slug are higher, in CKB-KO and shCKB cells, as compared to PC3 wild type (WT) cells. **P<0.01, n.s.: P>0.05, not significant, from 2-sided Student t-test. (B) Assessed by RT-qPCR, epithelial gene Occludin is induced by 24 h treatment of 15uM MK-2206 in the 4 indicted cell lines. Its basal expression is higher in PC3-parental and shScram control cells than in CKB-KO and shCKB cells. (C) As assessed by RT-qPCR, AKT inhibitor Perifosine upregulated epithelial genes and downregulated mesenchymal genes in shCKB cells. (D) Focus formation of PC3-shCKB cells treated with DMSO, 3uM MK-2206 and 10uM Perifosine for 12 days. These experiments have been repeated twice, which has yielded same conclusions. Results from a representative experiment are shown.

Supplementary Figure S5. Additional representative immunofluorescent images for Figure 8E. GFP-AKT-PH fusion protein (green), Flag-CKB full length (FL) or truncation protein (red) and nuclei (blue). White arrows indicate the PC3 cells transfected with the corresponding CKB full length or truncated cDNA constructs. Results from quantitative analysis of these and other images using Image J software are shown in Supplementary Figure S6A.

Supplementary Figure S6. Effects of CKB cDNA fragments in AKT membrane localization, focus formation and migration of PC3 cells. (A) Quantitative results from image analysis for the experiment in Figure 8E. PC3 cells expressing GFP-AKT-PH fusion protein were transfected with plasmids of Flag-CKB full length or fragments. Expression of GFP and CKB cDNA were indicated by green and red IF signals, respectively. Green signals for GFP-AKT-PH proteins in different cellular compartments of CKB transfected cells (red, n=3) or untransfected cells (not red, n=5) were quantified. The ratios of GFP signals on cell membrane vs. cytoplasm were calculated and plotted on Y-axis as Means + SD. (B) Additional representative images and quantitative results from image analysis for the experiment in Figure 9D. Green signals for GFP-AKT-PH proteins in different cellular compartments of CKB-84aa transfected cells (red, n=8) or untransfected cells (not red, n=11) were quantified. The ratios of GFP signals on cell membrane vs. cytoplasm were calculated and plotted on Y-axis as Means + SD. (C) Image analysis and quantitative results from Image J software for the focus formation experiment in Figure 9E. (D) Image analysis and quantitative results from Image J software for the migration experiment in Figure 9F.

Supplementary Figure S7. Co-evolution analysis and docking simulation of AKT and CKB interaction. (A) The top 20 coupling residue pairs for AKT and CKB using co-evolution methods DCA (left) and RaptorX (right), which could be possible interacting residues in the protein-protein recognition. The integral numbers represent positions of amino acids (aa) in the PH domain of AKT (aa1-110) (in dark red), and those in the 84aa fragment of CKB (aa298-381) (in magenta). (B-D) Three candidate binding models for AKT-CKB predicted from docking simulations. The PH domain of AKT (aa1-110) was colored in dark red, while the 84aa fragment of CKB (aa298-381) was colored in magenta.

Supplementary Table 1. Primer sequences

## Appendix Supplementary materials

Image, application 1

Image, application 2
