# Supplementary material for: CKB inhibits epithelial-mesenchymal transition and prostate cancer progression by sequestering and inhibiting AKT activation
Source: Neoplasia. 2021 Oct 24;23(11):1147–65. doi: 10.1016/j.neo.2021.09.005 (PMC8551525; doi:10.1016/j.neo.2021.09.005)
Supplement: Supplementary file 2 [file mmc2.pdf]

## 10 Figures and 7 Supplementary Figures

Figure 1

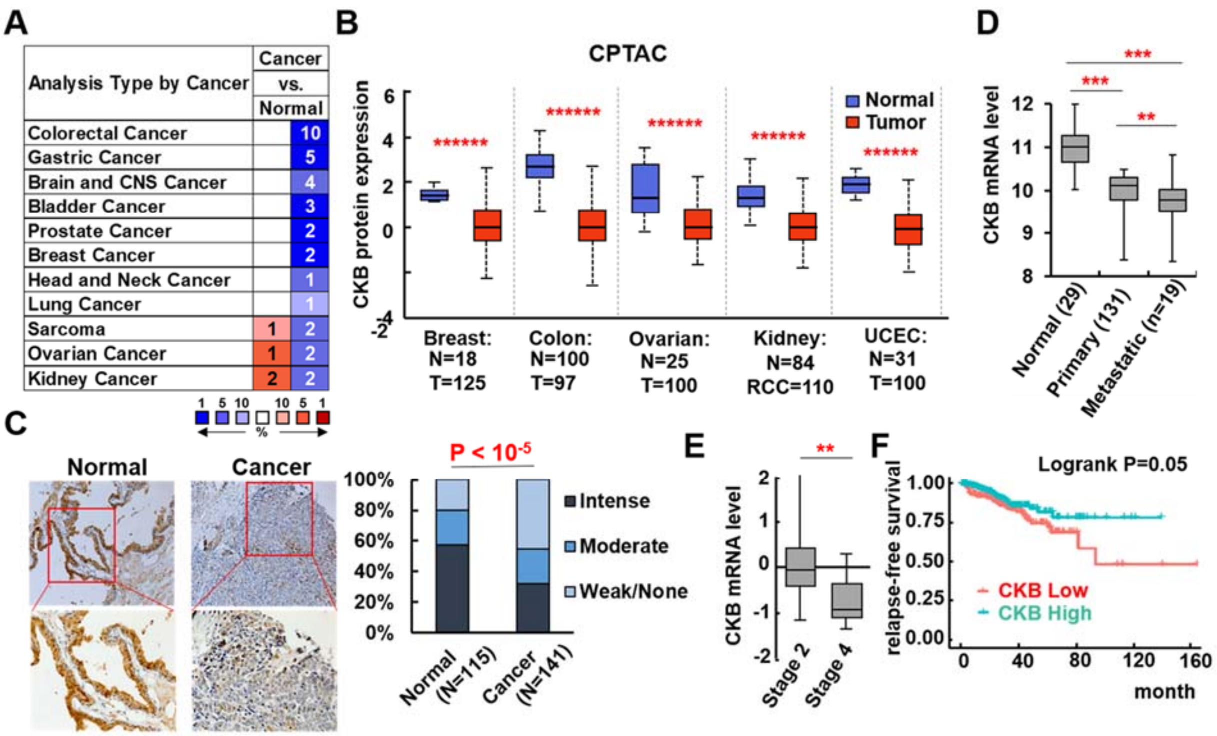


**Figure 1. CKB is downregulated in human solid tumors, which is associated with poor prognosis.**

**(A)** CKB mRNA expression in normal vs tumor comparisons of various cancer types from Oncomine.org website. Significance thresholds are: $P<=1x10^{-4}$, fold change >=2, and gene rank top 10%. Red signifies over-expression and blue represents under-expression in tumors. Intensities of color signify the best ranks of CKB in the analyses. The numbers represent the numbers of analyses that meet the thresholds. **(B)** CKB protein expression in normal and tumor samples, and the P values, were from the CPTAC database, obtained through UALCAN. **(C)** CKB protein expression was analyzed in prostate normal and tumor tissue microarray by immunohistochemistry. Staining was scored for each sample, and percentage of weak, moderate or intense CKB staining in normal and tumor samples was shown on the right. P value was from Chi Square Test. **(D-F)** CKB mRNA expression was analyzed in Taylor_Prostate dataset[45] **(D)**, and in TCGA prostate cancer dataset for tumor stage (stage 2, n=186 vs stage 4, n=11) **(E),** and biochemical relapse (BCR) free survival time **(F)**. **P<0.01, ***P<0.001 were from two-sided Student t-test. In KM survival analysis (F), patients were categorized as CKB Low or High based on the ROC curve method, and the P value was from Logrank test (GraphPad Prism).

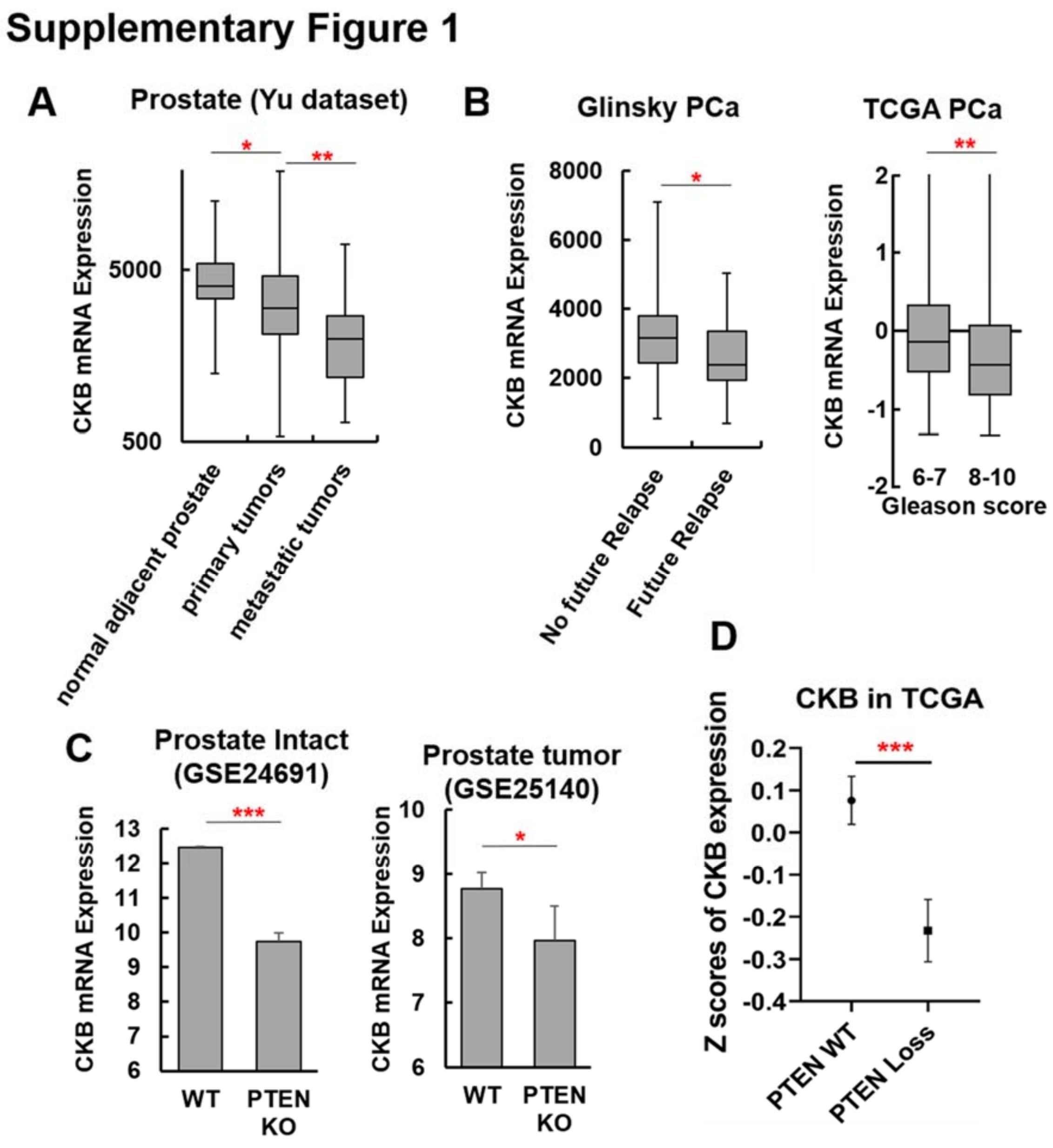


**Supplementary Figure S1. CKB is downregulated in prostate tumors in patients and mouse models.**

**(A)** CKB mRNA expression are analyzed in normal adjacent prostates (n=63), prostate primary tumors (n=65) and metastatic tumors (n=25) in Yu dataset (GSE6919). **(B)** CKB mRNA expression are analyzed in prostate cancer patient samples with (n=38) or without (n=42) future relapse (Glinsky dataset), or with different Gleason grades in TCGA dataset (Gleason score 6-7, n=291 vs Gleason score 8-10, n=207) (TCGA data through cBioPortal in July 2020). **(C)** Mouse CKB mRNA levels are analyzed in two PTEN knockout prostate cancer datasets as indicated. **(D)** CKB expression in PTEN-WT vs PTEN-loss patient tumors in the TCGA prostate cancer dataset (downloaded from cBioportal). PTEN mutations or loss of one or both alleles are considered PTEN-loss. *P<0.05, **P<0.01, ***P<0.001, from Student t-test.

## Supplementary Figure 2

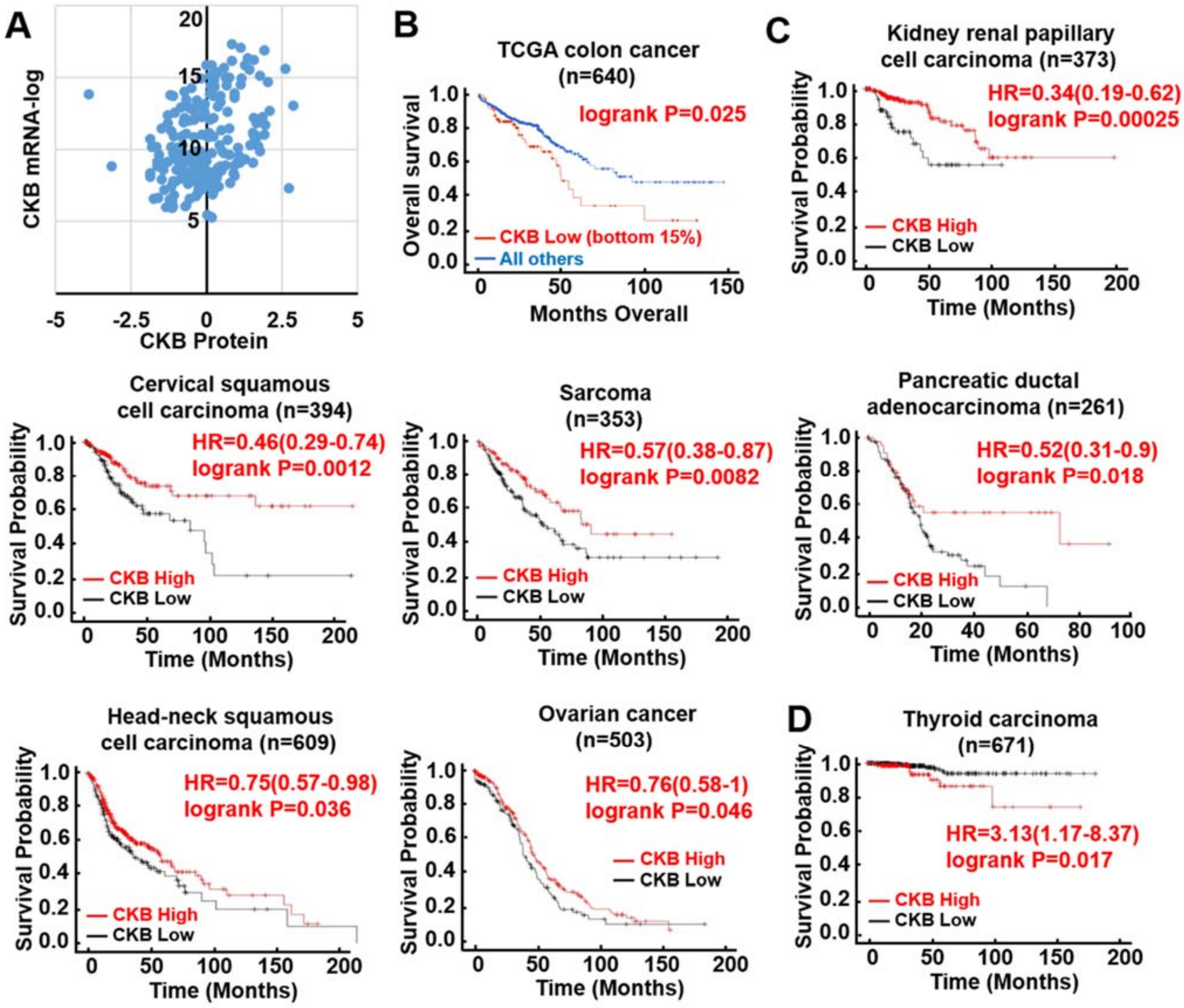


**Supplementary Figure S2. Patients with lower CKB expression correlate with poor overall survival.**

**(A)** CKB mRNA-protein correlation in 287 human tumors that have both CKB mRNA levels (TCGA) and protein levels (CPTAC) (R=0.397, P=2.97E-12). **(B)** Kaplan-Meier curves comparing TCGA colon cancer patients with altered CKB expression (15% samples with lower CKB expression, Z score < -0.65) vs all other patients (accessed through cBioPortal in July 2020). **(C-I)** Kaplan-Meier curves were shown for 7 solid tumor types where the Logrank P values were <0.05 for the association of CKB expression with survival of cancer patients (either positively or negatively). Six of seven cancer types show a negative correlation. CKB expression was defined as high or low in each RNA-seq datasets based on the best split. Plots and statistic data were downloaded from KMPlot (http://kmplot.com/analysis/index.php?p=service&cancer=pancancer_rnaseq).

Figure 2

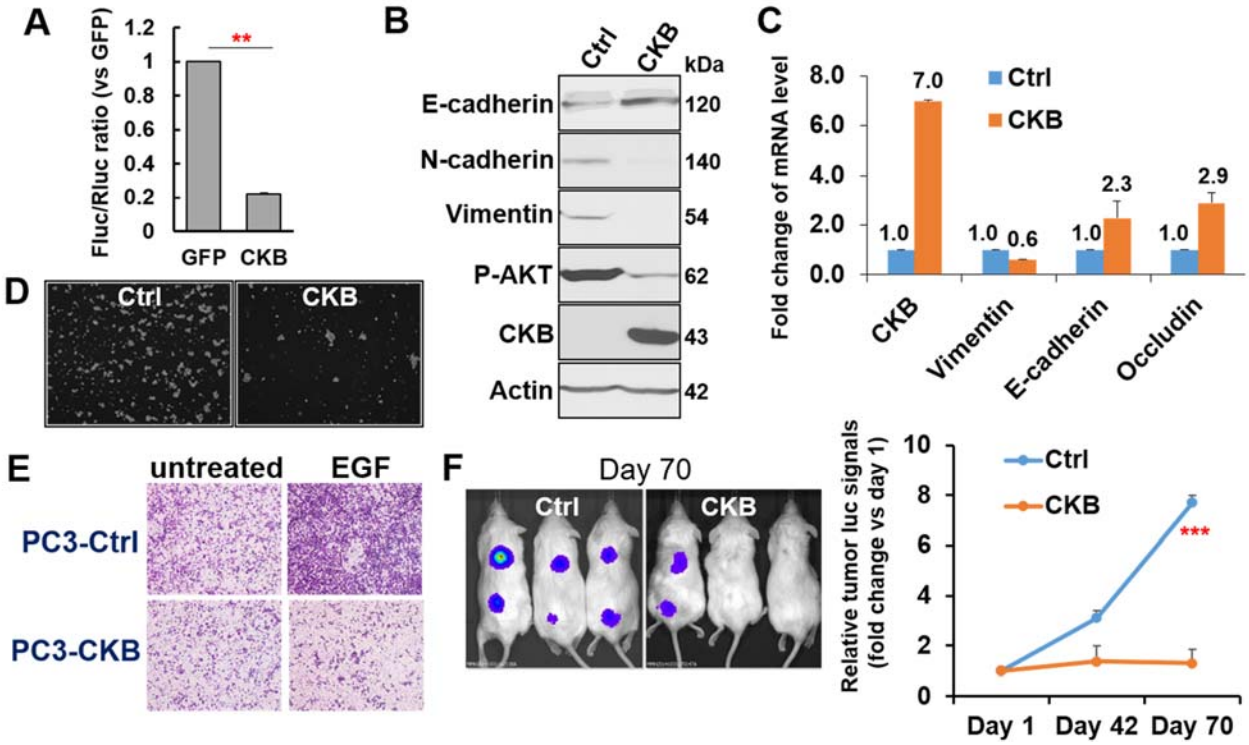


**Figure 2. CKB overexpression inhibits EMT, migration and tumor growth of prostate cancer cells.**

**(A)** Luciferase activity in 293T cells 36 hr after transfection with GFP (control, Crtl) or CKB cDNA plasmid, together with a Vimentin promoter firefly luciferase (Fluc) reporter and a renilla luciferase (Rluc) control construct. Relative fold change of the Fluc/Rluc ratio was calculated and plotted as means ± SD. **P<0.01 from triplicates. **(B)** Immunoblotting for indicated proteins in PC3 cells expressing GFP or CKB cDNA. **(C)** qPCR analysis of mRNA levels of indicated genes normalized to beta-actin in LN3 cells expressing GFP or CKB. **(D)** Anchorage-independent growth in a 24-well ultra-low attachment plate. **(E)** Cell migration as determined by Boyden chamber assay. Serum starved PC3-EV and -CKB cells were treated or not treated with 20 ng/ml of EGF for 8 hr, followed by migration assays. Quantifications from duplicate experiments are in Supplementary Figure S3A. **(F)** $1 \times 10^6$ PC3-luciferase cells expressing GFP (Ctrl) or CKB cDNA were implanted subcutaneously in male NOD/SCID mice (n=8 or 9 mice in either group). Xenograft tumor growth was monitored by bioluminescence imaging at day 1, day 42 and 70. Fold changes relative to day 1 were calculated and plotted as means ± SD. ***P<0.001 from comparing the control and CKB groups at day 70.

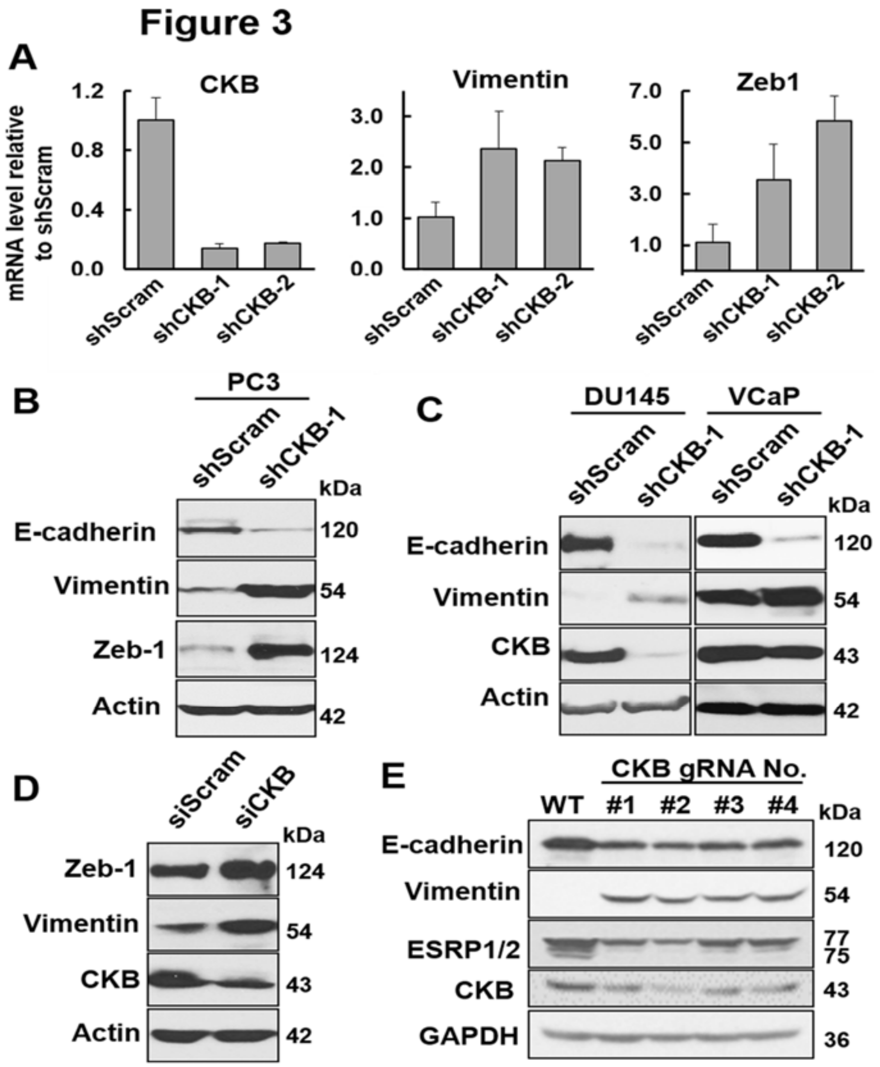


**Figure 3. Silencing CKB induces EMT markers in prostate cancer cells.**

**(A)** qPCR analysis of PC3 cells stably expressing scramble control shRNA or two independent CKB shRNAs (shCKB-1 and shCKB-2). Relative fold change was calculated and plotted as means ± SD. *P<0.05 comparing shScram to either shCKB group. **(B)** Immunoblotting of whole cell lysates of PC3 cells stably expressing scramble control shRNA or CKB shRNA. **(C)** Immunoblotting in DU145 and VCaP cells expressing control shScram or shCKB. **(D)** Immunoblotting of whole cell lysates of DU145 cells transfected with control siRNA or CKB siRNA. **(E)** Immunoblotting of PC3 parental cells (WT) and 4 pools of PC3 cells transfected with 4 different Cas9-gRNA plasmids targeting CKB. These PCR and immunoblotting experiments have been repeated three times with comparable results, and so one representative experiment is presented.

Figure 4

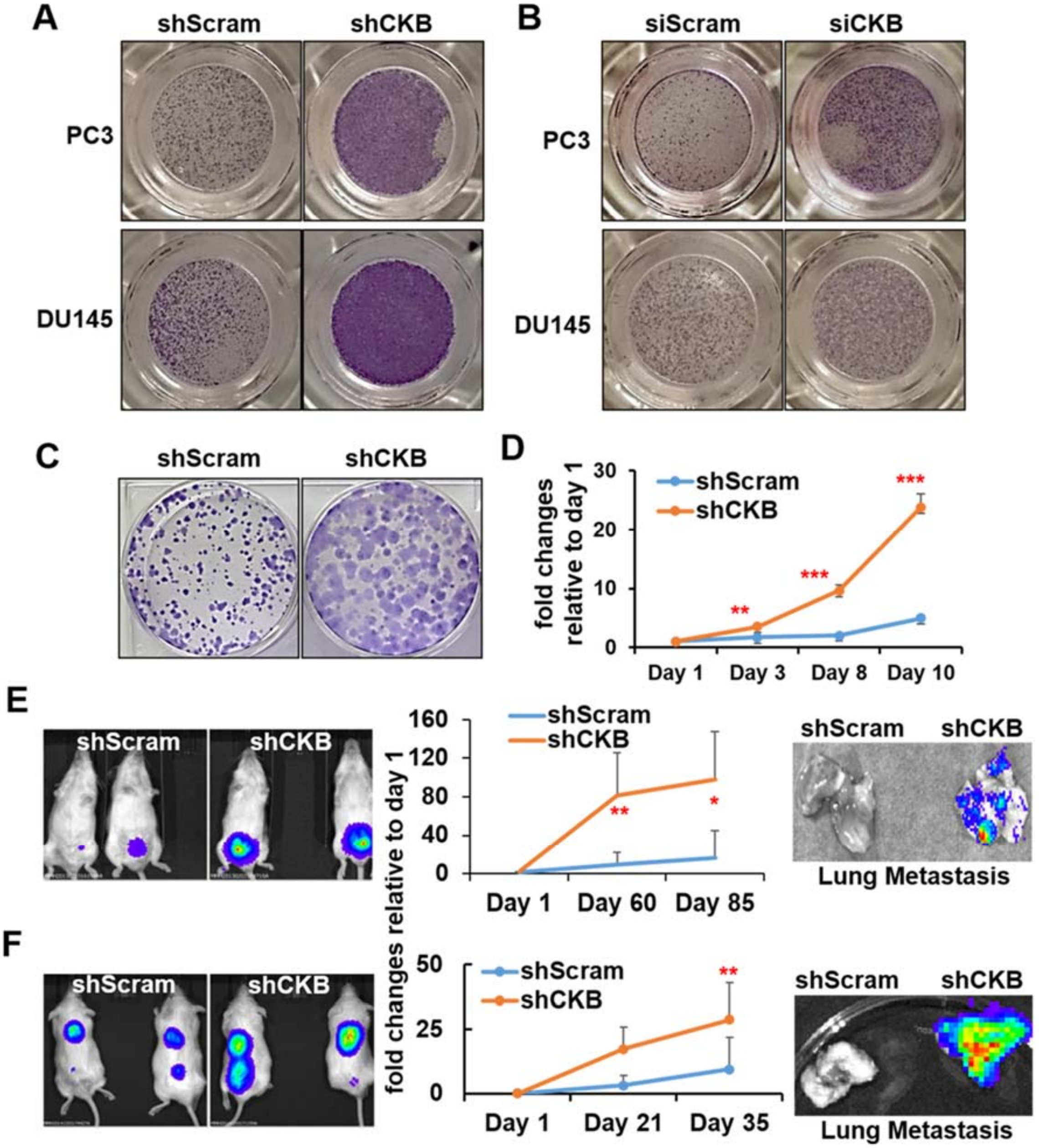


**Figure 4. Silencing CKB promotes prostate cancer cell migration and focus formation *in vitro*, as well as primary tumor growth and lung metastasis *in vivo*.**

**(A)** Cell migration as determined by Boyden chamber assay for PC3 and DU145 cells stably expressing control or CKB shRNA. Representative pictures from triplicate experiments are shown. **(B)** Boyden chamber cell migration assay for PC3 and DU145 cells transfected with control siRNA or CKB siRNA. Representative pictures from triplicate experiments are shown. Quantifications are in Supplementary Figure S3B. **(C)** Focus formation of PC3 cells stably expressing control or

CKB shRNA. Representative pictures from duplicate experiments are shown. Additional pictures are in Figure S3C. **(D)** Relative cell growth rate of PC3 cells expressing control shRNA (shScram) or shCKB-1 in culture medium supplied with low % of FBS (1.25%), at day 1, 3, 8 and 10 (quadruplicates). Relative fold changes to day 1 were calculated and plotted as Means ± SD. *P<0.05, **P<0.01, ***P<0.001 from comparing shScram with shCKB-1 cells at each time points (quadruplicates). **(E)** PC3 cells labelled with luciferase and expressing either shScram or shCKB-1 were implanted into prostates of NOD/SCID mice (shScram n=5, shCKB-1 n=4 mice). Fold changes of bioluminescence readings were calculated and plotted as Means ± SD (middle). Representative bioluminescence images of mice before sacrifice at day 85 (left) and *ex vivo* images for lung metastasis after sacrifice (right) are shown. **(F)** DU145 cells labelled with luciferase and expressing either shScram or shCKB-1 were implanted subcutaneously in NOD/SCID mice (shScram n=8, shCKB-1 n=8 mice). Fold changes of bioluminescence readings were calculated and plotted as Means ± SD (middle). Representative bioluminescence images of mice before sacrifice at day 35 (left) and *ex vivo* images for lung metastasis after sacrifice (right) are shown. *P<0.05, **P<0.01 comparing shScram with shCKB-1 group at the indicated time points (E and F).

## Supplementary Figure 3

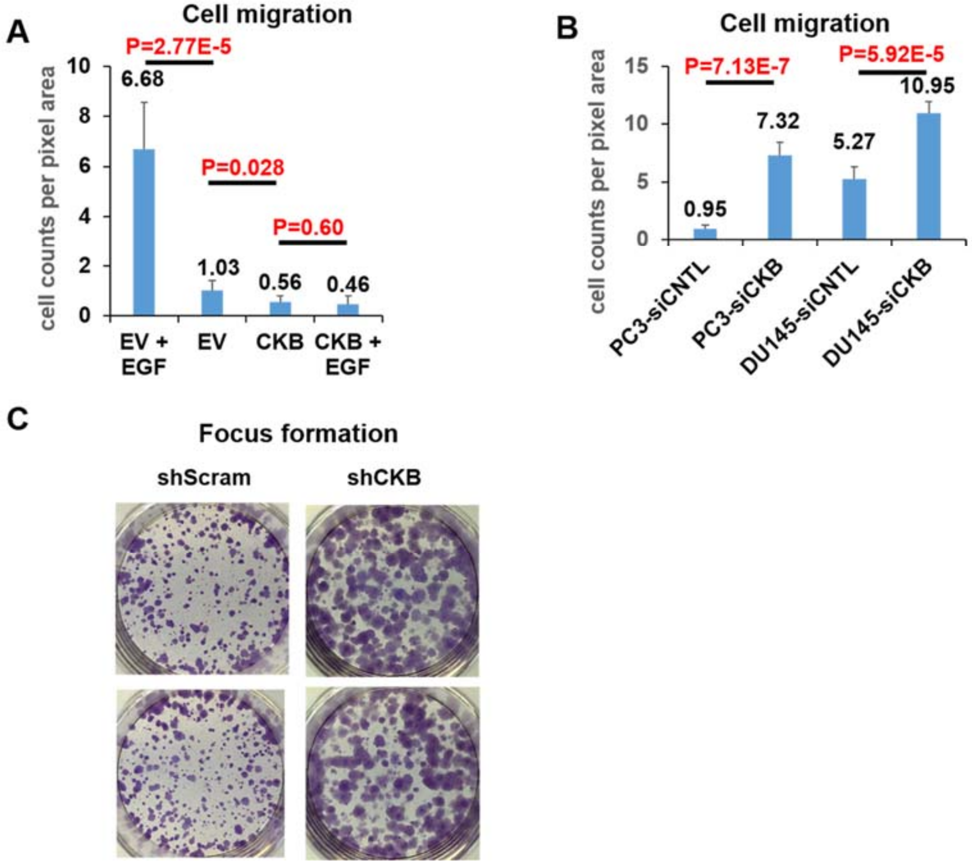


**Supplementary Figure S3. CKB overexpression ablates EGF-induced migration in PC3 cells, while its silencing promotes migration and colony formation.**

**(A)** Quantitative data from image analysis using Image J software on the migration experiments (triplicates) in Figure 2E. **(B)** Quantitative data from image analysis using Image J software on the migration experiments (triplicates) in Figure 4B. **(C)** Images of focus formation of PC3-shScram and shCKB cells in 6-well plates (duplicates). The sparsely seeded cells were grown for 12 or 14 days with fresh media replenishment every 5-6 days, followed by fixation and crystal violet staining.

Figure 5

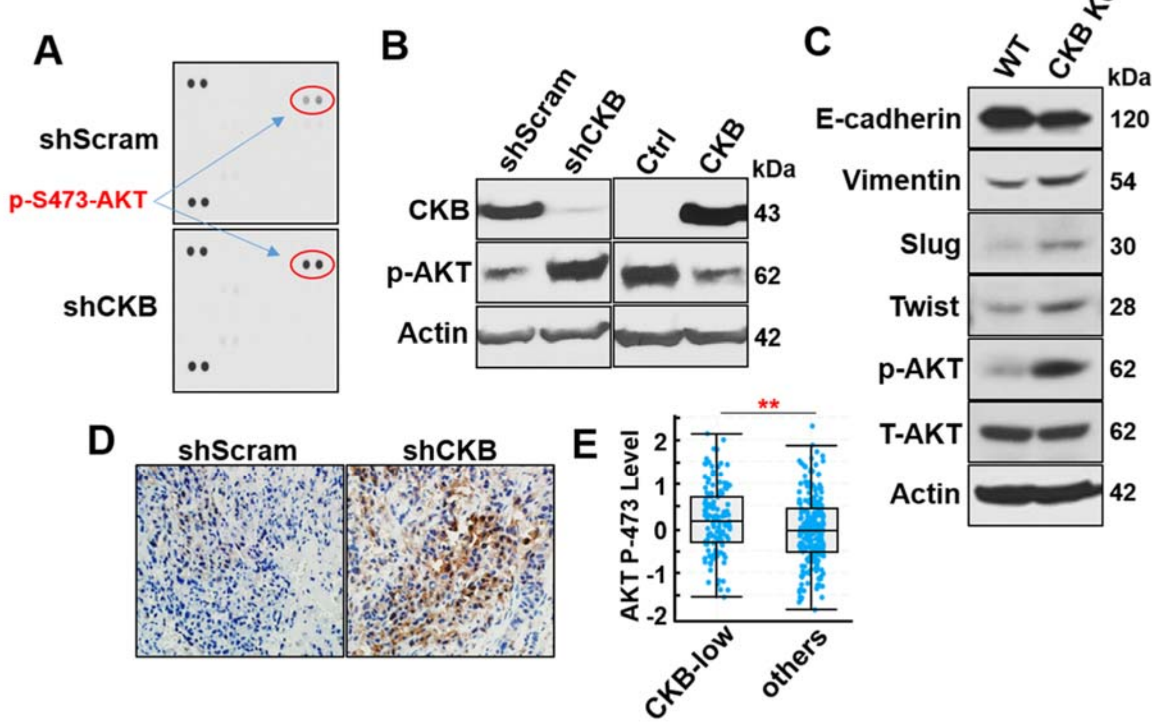


**Figure 5. CKB silencing induces AKT-S473 phosphorylation, consistent with their correlation in patient samples.**

**(A)** Whole cell lysate from PC3 cells expressing either shScram or shCKB-1 was tested on a human phospho-kinase array (R&D Systems). Red circles show duplicate spots with signals from p-S473-AKT antibody. **(B)** Immunoblotting for p-S473-AKT, CKB and Beta Actin in DU145 cells expressing shScram or shCKB-1 (left), and PC3 cells carrying empty vector control (EV, Ctrl) or CKB cDNA (right). **(C)** Immunoblotting for E-Cadherin, Vimentin, Slug, Twist, p-S473-AKT, AKT and beta-Actin in PC3 parental (WT) and CKB knockout cells. **(D)** Immunohistochemistry analysis of p-S473-AKT in PC3-shScramble and PC3-shCKB xenografts. **(E)** p-S473-AKT levels in TCGA primary prostate tumors, as analyzed by RPPA (accessed through cBioportal), comparing samples with low CKB mRNA expression (Z-score <-0.5) with all other samples. The p-S473-AKT plot and P value (P<0.01) were obtained from cBioPortal.

Figure 6

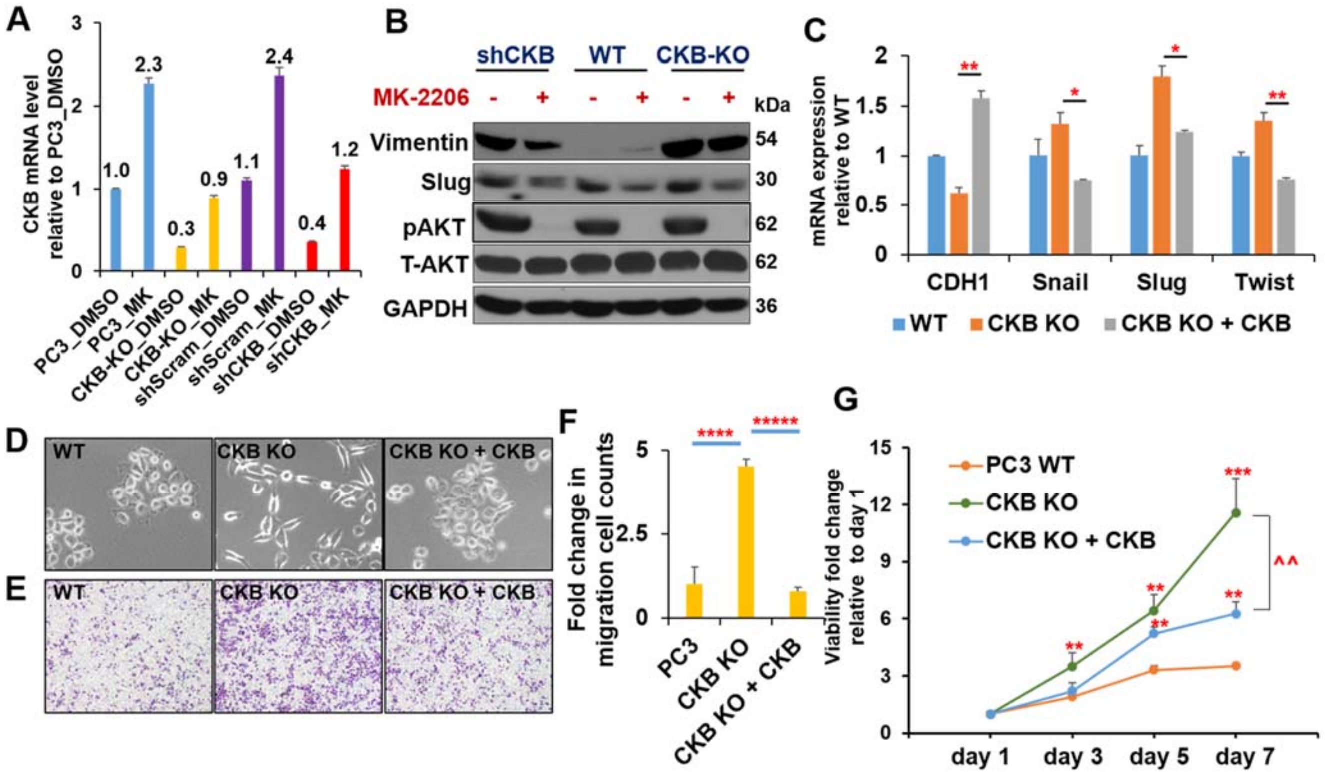


**Figure 6. AKT inhibitor and CKB overexpression reverse EMT induced by CKB suppression.**

**(A)** RT-qPCR result of CKB expression in the indicated 4 cell lines treated with DMSO or 15uM AKT inhibitor MK-2206 for 24hr. Expression pattern for epithelial marker Occludin from these experiments is in Figure S4B. **(B)** Parental PC3 cells (wild type, WT), PC3 with CKB CRISPR-Cas9 knockout (CKB KO), or PC3 with CKB knockdown (shCKB-1) were treated with DMSO or 3uM of AKT inhibitor MK-2206 for 48h. Immunoblots for indicated proteins were shown. **(C)** qPCR analysis for indicated genes normalized to beta-Actin in PC3 parental cells (WT), CKB knockout cells (CKB KO), and CKB-knockout cells re-expressing CKB cDNA (CKB KO + CKB). $^*P<0.05$, $^{**}P<0.01$ comparing CKB KO to CKB KO + CKB. **(D)** Cell morphology were monitored by phase-contrast microscopy in PC3 WT, CKB KO and CKB KO + CKB cells. **(E)** Boyden chamber migration assay for PC3 WT cells, PC3 CKB KO cells and PC3 CKB KO + CKB cells. **(F)** Quantitative results of the migrations in E, based on triplicates. $^{***}P<0.001$, $^{****}P<1x10^{-4}$, $^{*****}P<1x10^{-5}$. **(G)** Alamar blue cell proliferation assay for PC3 WT, CKB KO and CKB KO + CKB cells cultured in media with reduced FBS (1.25%) at day 1, 3, 5 and 7 (quadruplicates). $^{**}P<0.01$, $^{***}P<0.001$ comparing CKB KO cells, CKB KO + CKB cDNA cells with WT cells at corresponding time points. ^^P<0.01 comparing CKB KO with CKB KO + CKB cells at day 7. These experiments have been repeated at least twice, which has yielded same conclusions. Results from a representative experiment are shown.

## Supplementary Figure 4

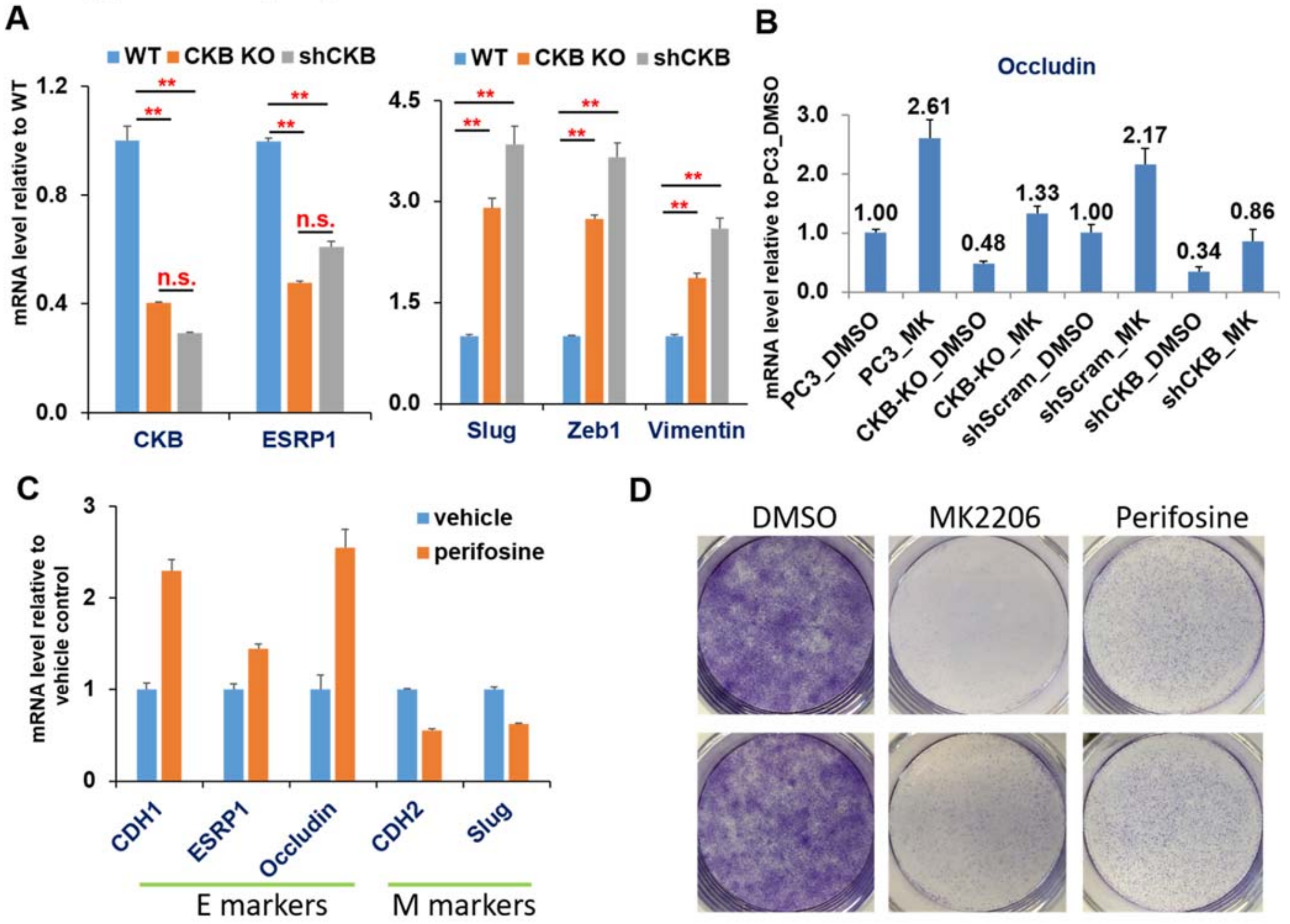


**Supplementary Figure S4. AKT inhibitors MK-2206 and Perifosine inhibit EMT gene expression and focus formation of PC3 CKB-KO and shCKB cells.**

**(A)** Assessed by RT-qPCR, CKB and epithelial gene ESRP1 are lower, while mesenchymal genes Vimentin, Zeb1 and Slug are higher, in CKB-KO and shCKB cells, as compared to PC3 wild type (WT) cells. **P<0.01, n.s.: P>0.05, not significant, from 2-sided Student t-test. **(B)** Assessed by RT-qPCR, epithelial gene Occludin is induced by 24hr treatment of 15uM MK-2206 in the 4 indicted cell lines. Its basal expression is higher in PC3-parental and shScram control cells than in CKB-KO and shCKB cells. **(C)** As assessed by RT-qPCR, AKT inhibitor Perifosine upregulated epithelial genes and downregulated mesenchymal genes in shCKB cells. **(D)** Focus formation of PC3-shCKB cells treated with DMSO, 3uM MK-2206 and 10uM Perifosine for 12 days. These experiments have been repeated twice, which has yielded same conclusions. Results from a representative experiment are shown.

## Figure 7

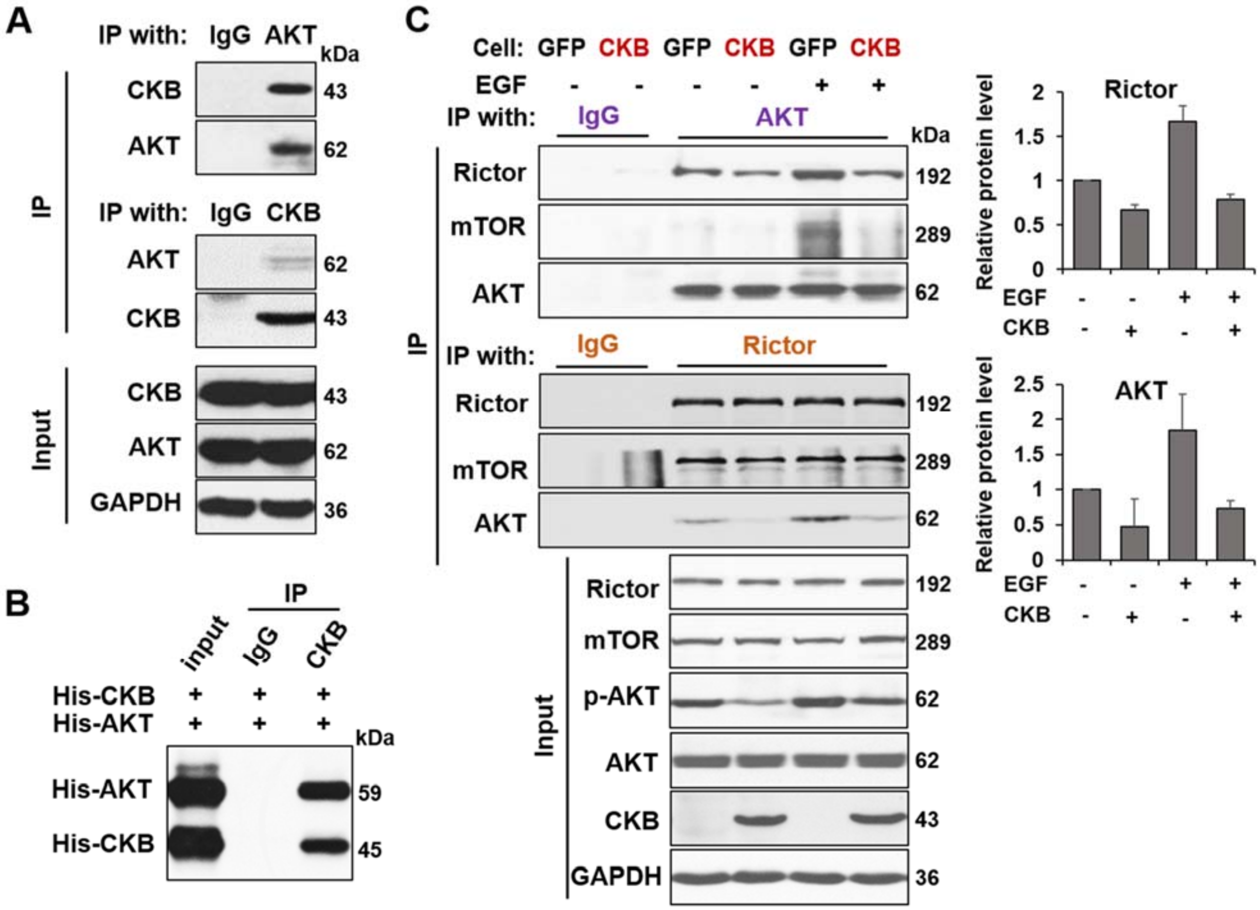

**Figure 7. CKB interacts with AKT and inhibits AKT activation.**

**(A)** CKB and AKT proteins could reciprocally co-immunoprecipitate (co-IP) each other from LNCaP cells. **(B)** AKT protein was immunoprecipitated by CKB antibody in a mixture of recombinant His-tagged AKT and His-tagged CKB proteins. **(C)** PC3-GFP and PC3-CKB cells were treated with or without 100ng/ml EGF for 5min. Endogenous AKT was immunoprecipated from these two cell lines using AKT Ab, followed by immunoblotting for Rictor, mTOR and AKT (top). Conversely, endogenous Rictor was immunoprecipated using Rictor Ab, then immunoblot for AKT, mTOR and Rictor (middle). Immunoblotting of the input whole cell lysates was shown in bottom. Fold changes of Rictor and AKT protein levels in the IP samples are plotted, relative to the sample without EGF and without CKB overexpression. The quantification is based on measurements from two independent experiments, using ImageJ software.

# Figure 8

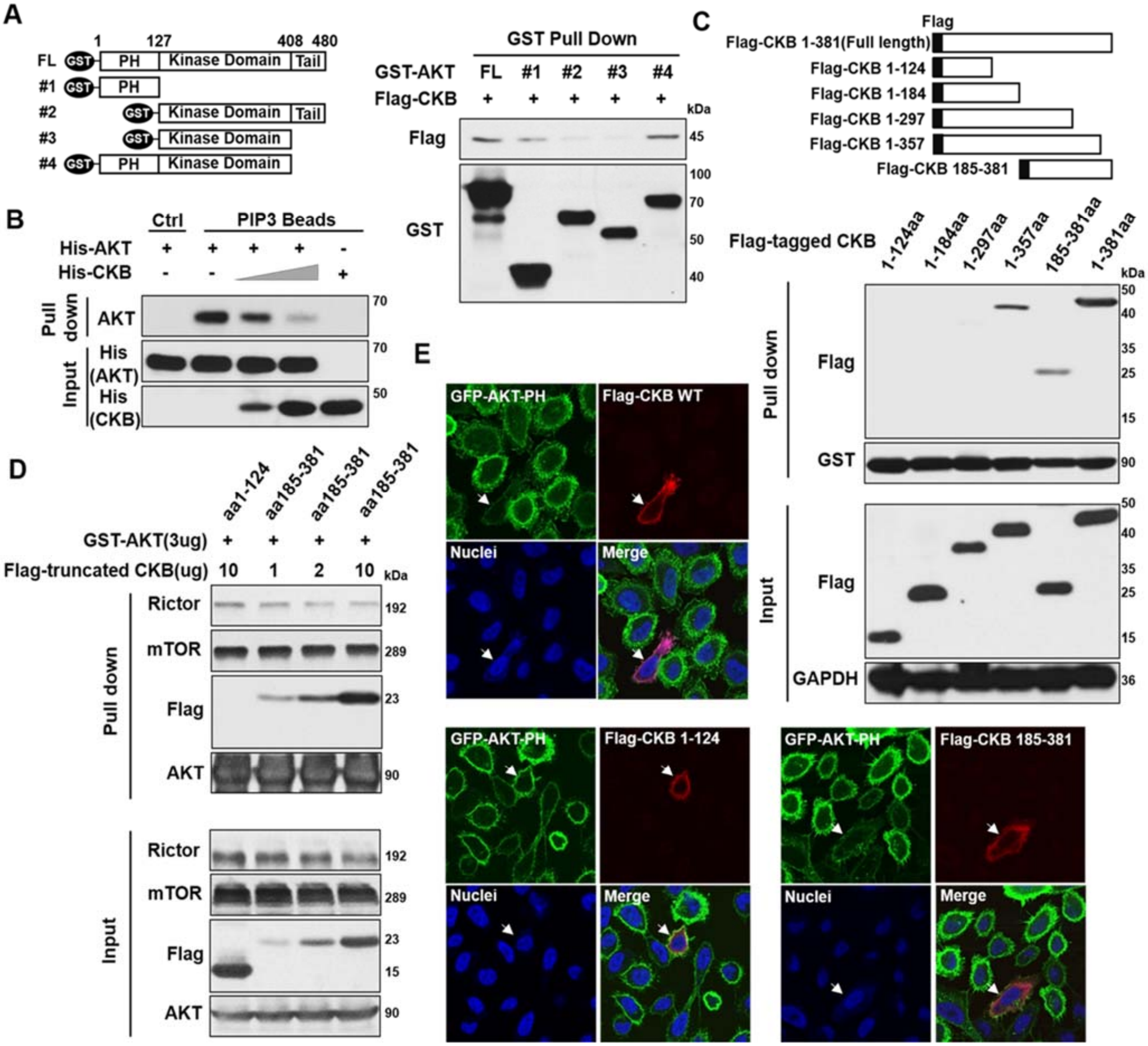


**Figure 8. CKB interacts with AKT PH domain through its C-terminal.**

**(A)** Schematic of GST-tagged AKT full-length (FL) and truncation mutants (left). 293T cells were transfected with cDNA vectors for GST-tagged AKT FL or truncations, together cDNA plasmid for Flag-tagged CKB FL protein. GST-tagged AKT proteins were immunoprecipitated by glutathione sepharose beads from co-transfected cells, followed by immunoblotting for Flag and GST (right). **(B)** PIP3 coated agarose beads were incubated with purified His-tagged AKT (2ug) and/or His-tagged CKB (0, 1 or 2ug) as indicated. PIP3 binding proteins were pulled down. Immunoblots for AKT and CKB were shown. **(C)** Schematic of Flag-tagged CKB FL and truncation mutants (top). GST-tagged AKT proteins were pull down by glutathione sepharose beads from 293T cells co-transfected with plasmids for GST-tagged AKT FL and Flag-tagged CKB FL or truncations as indicated (middle). Immunoblots on input whole cell lysates are in the bottom. **(D)** 293T cells were

co-transfected with GST-tagged AKT vector and indicated amounts of Flag-tagged CKB truncation vectors. GST-tagged AKT proteins were pull down by glutathione sepharose beads from these cells. Immunoblots for Rictor, mTOR, Flag and AKT were analyzed. **(E)** Representative immunofluorescence images for GFP-AKT-PH fusion protein (green), Flag-CKB FL protein or Flag-CKB truncations (red) and nuclei (blue). PC3 cells expressing GFP-AKT-PH were transfected with Flag-CKB plasmids. White arrows indicate the PC3 cells transfected with the corresponding CKB full length or truncated cDNA constructs. Untransfected cells in the same wells serve as controls. Additional representative images are in Supplementary Figure S5. Quantifications of GFP-AKT-PH signal ratios on membrane vs. cytoplasm in multiple untransfected and transfected cells are presented in Figure S6A. These immunoblotting and IF experiments have been repeated twice, which has yielded same conclusions. Results from a representative experiment are shown.

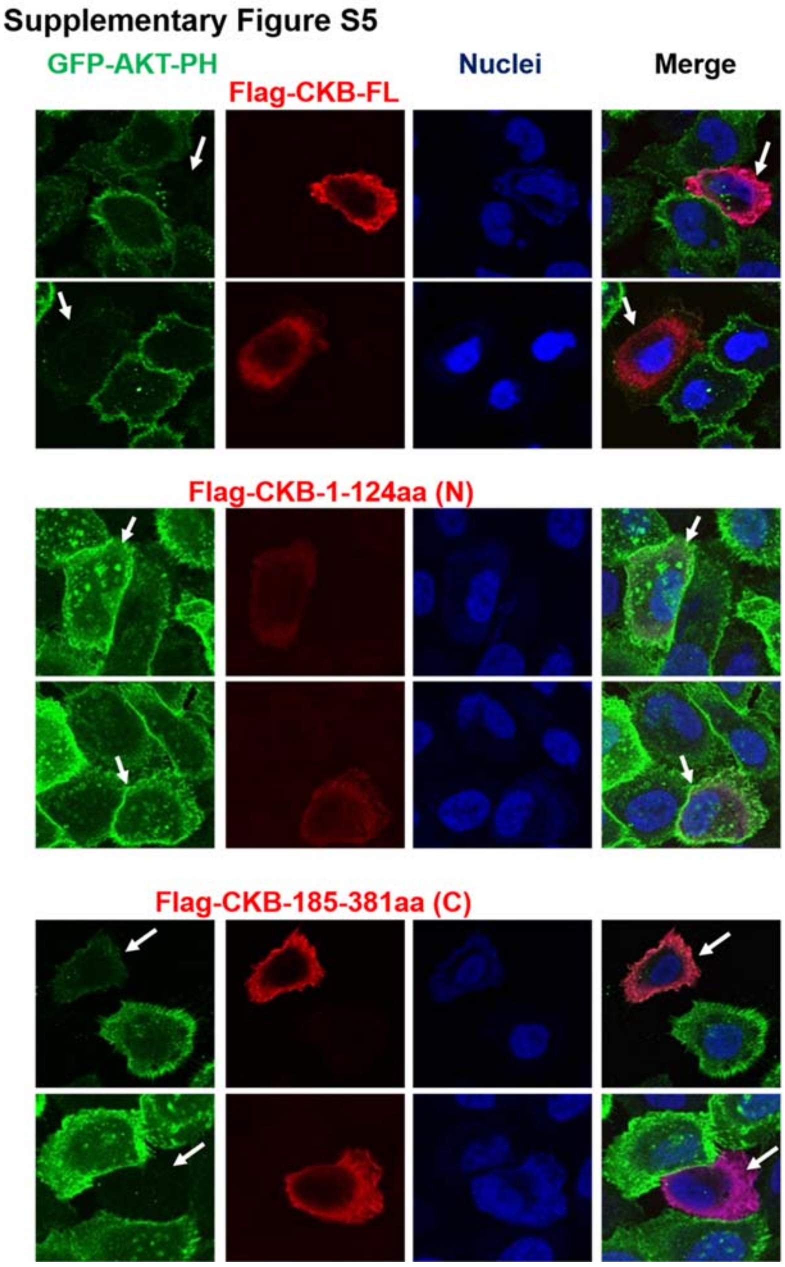


**Supplementary Figure S5. Additional representative immunofluorescent images for Figure 8E.** GFP-AKT-PH fusion protein (green), Flag-CKB full length (FL) or truncation protein (red) and nuclei (blue). White arrows indicate the PC3 cells transfected with the corresponding CKB full length or truncated cDNA constructs. Results from quantitative analysis of these and other images using Image J software are shown in Supplementary Figure S6A.

## Figure 9

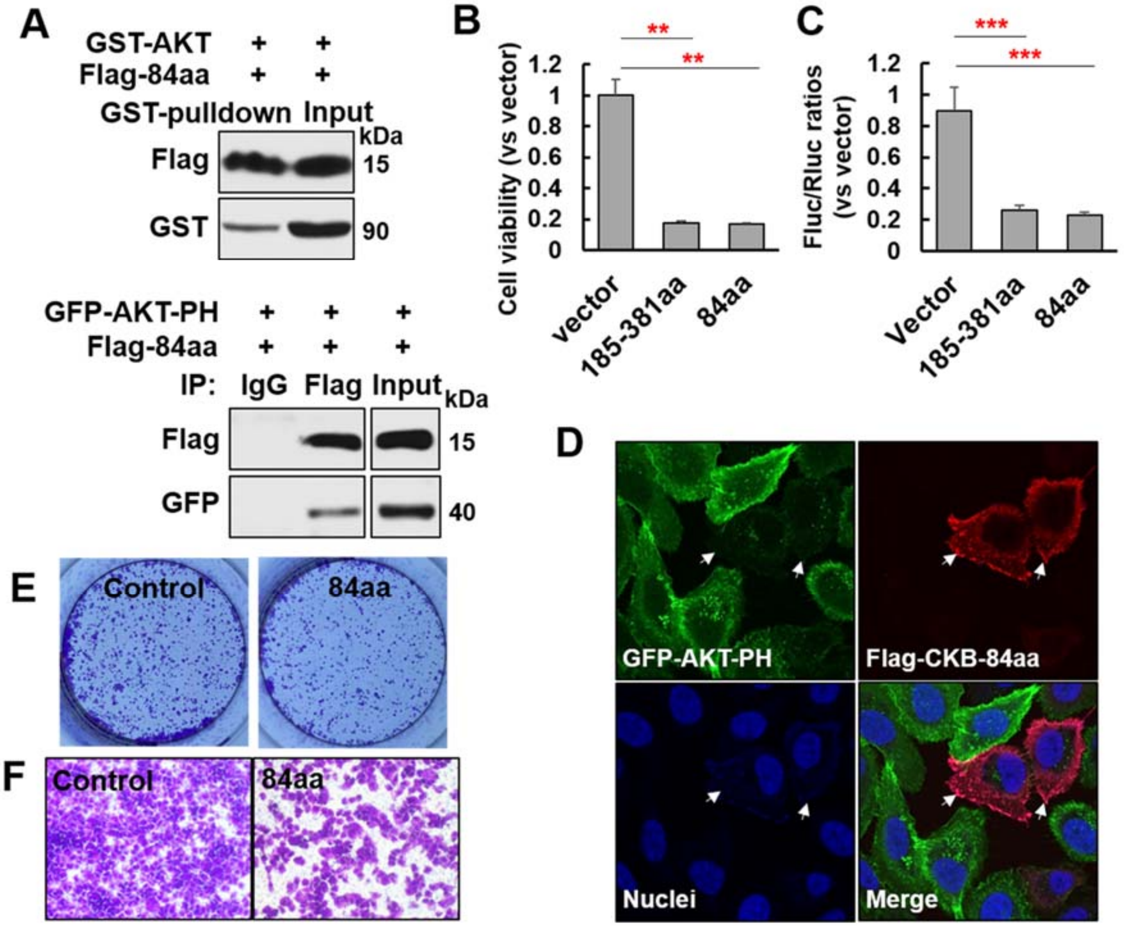


**Figure 9. C-terminal 84aa fragment of CKB protein inhibits Vimentin promoter activity, focus formation and migration of PC3 cells.**

**(A)** (Top) GST-tagged AKT proteins were pull down by glutathione sepharose beads from 293T cells co-transfected with plasmids for GST-tagged AKT FL and Flag-tagged CKB-84aa (298aa-381aa), as indicated. Immunoblots for Flag and GST were shown. (Bottom) Immunoprecipitation assay using anti-Flag antibody from 293T cells co-transfected with plasmids for GFP-AKT-PH and Flag-tagged CKB C-terminal 84aa fragment. Immunoblots for Flag and GFP were shown. **(B)** Cell proliferation assay measured by alamar blue in 293T cells transfected with Flag empty vector, Flag-tagged CKB-185aa-381aa or Flag-tagged CKB-298aa-381aa (84aa) plasmids. **(C)** Luciferase activity in lysates co-transfected with Flag vector, Flag-tagged CKB-185-381aa or Flag-tagged CKB-84aa plasmids, together with Vimentin promoter firefly luciferase reporter and pGL4.74 renilla luciferase plasmids in 293T cells. 24h later, cells were lysed for measuring luciferase activity. **$P<0.01$, ***$P<0.001$ from 2-sided Student t-test comparing either CKB construct to vector control (triplicates). **(D)** Representative immunofluorescence images for GFP-AKT-PH (green), Flag-CKB 84aa fragment (red) and nuclei (blue). PC3 cells expressingGFP-AKT-PH were transfected with plasmid for Flag-CKB-84aa. White arrows indicate the PC3 cells transfected with Flag-CKB-84aa construct. Untransfected cells in the same wells serve as controls. Quantifications of GFP-AKT-PH signal ratios of membrane vs. cytoplasm in untransfected (not red) and transfected (red) cells are presented in Figure S6B. **(E)** Focus formation assay of PC3

cells infected with lentivirus carrying either vector control or CKB-84aa. **(F)** Cell migration determined by Boyden chamber assay in PC3 cells infected with lentivirus carrying either vector control or CKB-84aa. Quantifications of focus formation and migrations (triplicates) are in Figure S6C-D. These experiments have been repeated at least twice, which has yielded same conclusions. Results from a representative experiment are shown.

**Supplementary Figure 6**

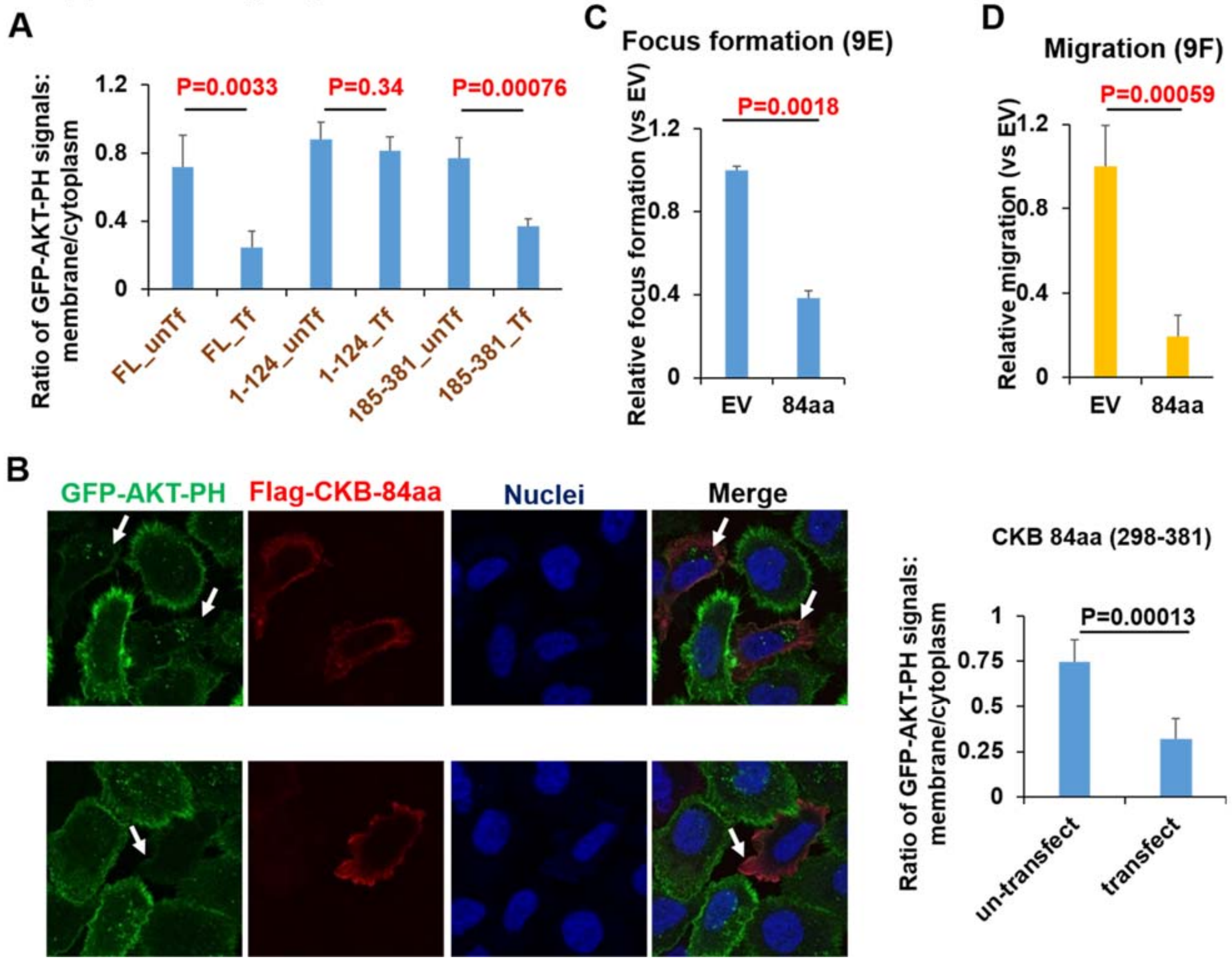


**Supplementary Figure S6. Effects of CKB cDNA fragments in AKT membrane localization, focus formation and migration of PC3 cells.**

**(A)** Quantitative results from image analysis for the experiment in Figure 8E. PC3 cells expressing GFP-AKT-PH fusion protein were transfected with plasmids of Flag-CKB full length or fragments. Expression of GFP and CKB cDNA were indicated by green and red IF signals, respectively. Green signals for GFP-AKT-PH proteins in different cellular compartments of CKB transfected cells (red, n=3) or untransfected cells (not red, n=5) were quantified. The ratios of GFP signals on cell membrane vs. cytoplasm were calculated and plotted on Y-axis as Means + SD. **(B)** Additional representative images and quantitative results from image analysis for the experiment in Figure 9D. Green signals for GFP-AKT-PH proteins in different cellular compartments of CKB-84aa transfected cells (red, n=8) or untransfected cells (not red, n=11) were quantified. The ratios of

GFP signals on cell membrane vs. cytoplasm were calculated and plotted on Y-axis as Means + SD. **(C)** Image analysis and quantitative results from Image J software for the focus formation experiment in Figure 9E. **(D)** Image analysis and quantitative results from Image J software for the migration experiment in Figure 9F.

## Supplementary Figure 7

**A** DCA coupling score and RaptorX probabilities

| AKT | CKB | Coupling Score | AKT | CKB | Probabilities |
|---|---|---|---|---|---|
| 103 | 354 | 0.50489 | 103 | 354 | 0.508734 |
| 107 | 354 | 0.49616 | 107 | 354 | 0.501918 |
| 60 | 254 | 0.49118 | 60 | 254 | 0.491181 |
| 103 | 350 | 0.46568 | 103 | 350 | 0.465951 |
| 106 | 350 | 0.46156 | 106 | 350 | 0.463352 |
| 106 | 354 | 0.45847 | 106 | 354 | 0.462031 |
| 103 | 357 | 0.42916 | 103 | 357 | 0.433663 |
| 407 | 88 | 0.41456 | 407 | 88 | 0.412002 |
| 100 | 361 | 0.39243 | 100 | 361 | 0.39622 |
| 57 | 254 | 0.35533 | 74 | 333 | 0.360048 |
| 107 | 358 | 0.35382 | 107 | 358 | 0.357396 |
| 74 | 333 | 0.34758 | 57 | 254 | 0.355177 |
| 103 | 361 | 0.3435 | 74 | 335 | 0.349771 |
| 110 | 354 | 0.34241 | 110 | 354 | 0.346297 |
| 102 | 350 | 0.34045 | 103 | 361 | 0.346046 |
| 74 | 335 | 0.3374 | 75 | 336 | 0.344875 |
| 407 | 91 | 0.33317 | 73 | 336 | 0.343577 |
| 75 | 336 | 0.33242 | 102 | 350 | 0.339732 |
| 73 | 336 | 0.3317 | 407 | 91 | 0.338899 |
| 96 | 361 | 0.33159 | 96 | 361 | 0.335582 |

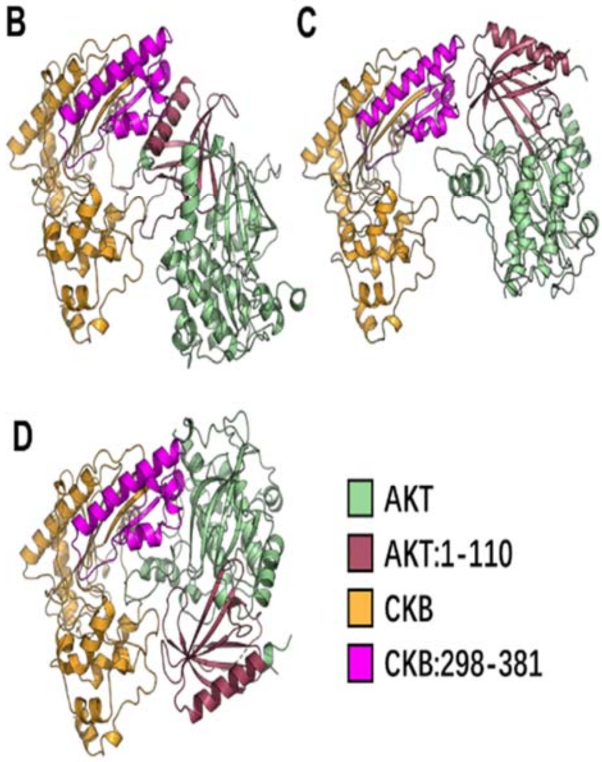


**Supplementary Figure S7. Co-evolution analysis and docking simulation of AKT and CKB interaction.**

**(A)** The top 20 coupling residue pairs for AKT and CKB using co-evolution methods DCA (left) and RaptorX (right), which could be possible interacting residues in the protein-protein recognition. The integral numbers represent positions of amino acids (aa) in the PH domain of AKT (aa1-110) (in dark red), and those in the 84aa fragment of CKB (aa298-381) (in magenta). (**B-D)** Three candidate binding models for AKT-CKB predicted from docking simulations. The PH domain of AKT (aa1-110) was colored in dark red, while the 84aa fragment of CKB (aa298-381) was colored in magenta.

# Figure 10

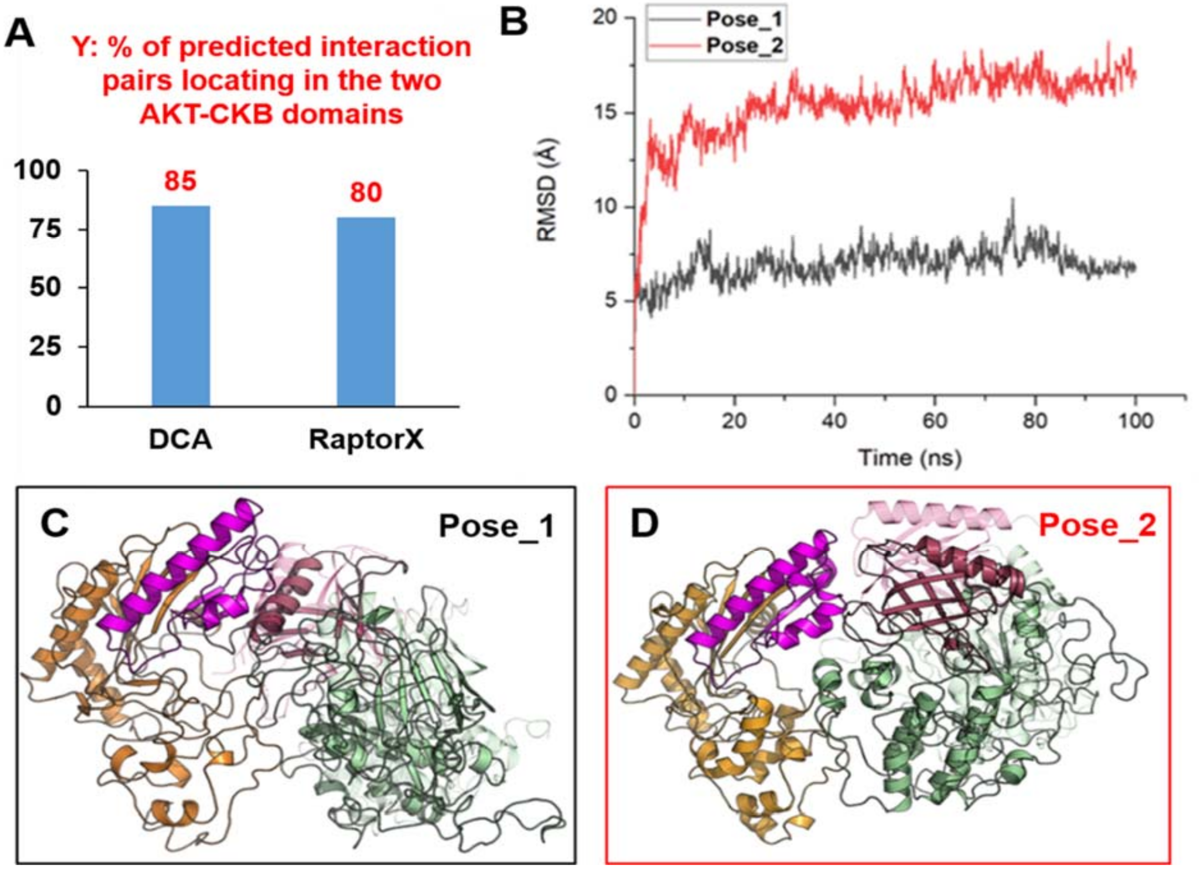


**Figure 10**. **AKT's PH domain and CKB's C-terminal 84aa fragment establish the major interaction interface of these two proteins in molecular dynamics simulations.**

**(A)** Potential interaction residues between AKT and CKB proteins were predicted using two different co-evolution techniques DCA and RaptorX, based on their amino acid sequences. Y axis: Percentage (%) of the top 20 predicted interaction amino acid pairs that reside in the PH domain of AKT and the C-terminal 84aa fragment of CKB. The top 20 residue pairs were listed in Supplementary Figure S3A. **(B)** The RMSD of Cα atoms of AKT and CKB in 100ns trajectory. RMSD (Root Mean Square Deviation) means that the structural variability between the structures with the initial structure in molecular dynamics simulation. A higher RMSD value means grater differences with initial structure. **(C) and (D)** The initial and final structures for the two binding modes before and after molecular dynamics simulations. The initial structure of AKT before molecular dynamics simulation is shown as transparent mode. The Pose_1 indicates the simulation results for binding pose illustrated in Figure 10C and the Pose_2 is for Figure 10D.
